# SARS-CoV-2 vaccines elicit durable immune responses in infant rhesus macaques

**DOI:** 10.1126/sciimmunol.abj3684

**Published:** 2021-06-15

**Authors:** Carolina Garrido, Alan D. Curtis, Maria Dennis, Sachi H. Pathak, Hongmei Gao, David Montefiori, Mark Tomai, Christopher B. Fox, Pamela A. Kozlowski, Trevor Scobey, Jennifer E. Munt, Michael L. Mallory, Pooja T. Saha, Michael G. Hudgens, Lisa C. Lindesmith, Ralph S. Baric, Olubukola M. Abiona, Barney S. Graham, Kizzmekia S. Corbett, Darin Edwards, Andrea Carfi, Genevieve Fouda, Koen K. A. Van Rompay, Kristina De Paris, Sallie R. Permar

**Affiliations:** 1 Duke Human Vaccine Institute, Duke University Medical Center, Durham, NC, USA.; 2 Department of Microbiology and Immunology, Center for AIDS Research, and Children’s Research Institute, School of Medicine, University of North Carolina at Chapel Hill, Chapel Hill, NC, USA.; 3 3M Corporate Research Materials Laboratory, Saint Paul, MN, USA.; 4 Infectious Disease Research Institute, Seattle, WA, USA.; 5 Department of Microbiology, Immunology and Parasitology, Louisiana State University Health Sciences Center, New Orleans, LA, USA.; 6 Department of Epidemiology, Gillings School of Global Public Health, University of North Carolina at Chapel Hill, Chapel Hill, NC, USA.; 7 Department of Biostatistics, Gillings School of Global Public Health, University of North Carolina at Chapel Hill, Chapel Hill, NC, USA.; 8 Vaccine Research Center, National Institute of Allergy and Infectious Diseases, National Institutes of Health, Bethesda, MA, USA.; 9 Moderna Inc., Cambridge, MA, USA.; 10 California National Primate Research Center, University of California, Davis, CA, USA.; 11 Cornell Weill Medical College, New York, NY, USA.

## Abstract

The inclusion of infants in the SARS-CoV-2 vaccine rollout is important to prevent severe complications of pediatric SARS-CoV-2 infections and to limit transmission and could possibly be implemented via the global pediatric vaccine schedule. However, age-dependent differences in immune function require careful evaluation of novel vaccines in the pediatric population. Toward this goal, we assessed the safety and immunogenicity of two SARS-CoV-2 vaccines. Two groups of eight infant rhesus macaques (RMs) were immunized intramuscularly at weeks 0 and 4 with stabilized prefusion SARS-CoV-2 S-2P spike (S) protein encoded by mRNA encapsulated in lipid nanoparticles (mRNA-LNP) or the purified S protein mixed with 3M-052, a synthetic TLR7/8 agonist in a squalene emulsion (Protein+3M-052-SE). Neither vaccine induced adverse effects. Both vaccines elicited high-magnitude IgG binding to RBD, amino-terminal domain, S1 and S2, ACE2 blocking activity, and high neutralizing antibody titers, all peaking at week 6. S-specific memory B cells were detected by week 4, and S-specific T cell responses were dominated by the production of IL-17, IFN-γ, or TNF-α. Antibody and cellular responses were stable through week 22. The immune responses for the mRNA-LNP vaccine were of a similar magnitude to those elicited by the Moderna mRNA-1273 vaccine in adults. The S-2P mRNA-LNP and Protein–3M-052-SE vaccines were well tolerated and highly immunogenic in infant RMs, providing proof of concept for a pediatric SARS-CoV-2 vaccine with the potential for durable immunity that might decrease the transmission of SARS-CoV-2 and mitigate the ongoing health and socioeconomic impacts of COVID-19.

## INTRODUCTION

Severe acute respiratory syndrome coronavirus 2 (SARS-CoV-2) has infected hundreds of millions of people worldwide and caused over 3.5 million deaths since its emergence in 2019. The need for safe and effective measures to limit transmission and mitigate public health and socioeconomic impacts of SARS-CoV-2 infection has prompted unprecedented vaccine development of promising candidates. Two mRNA vaccines, mRNA-1273 (Moderna) (1, 2) and BNT162b2 (Pfizer-BioNTech) (3, 4), are authorized for emergency use in the United States to prevent SARS-CoV-2 infection in adults. Both are safe and induce neutralizing antibodies (Abs) and up to 95% protection from disease. In addition, the Oxford/AstraZeneca adenovirus-based ChAdOx1 nCoV-19 (AZD1222) (5), the Johnson & Johnson Ad26.COV2.S vaccine (JNJ-78436735) (6), and the first protein-based SARS-CoV-2 vaccine, Novavax NVX-CoV2373, adjuvanted with saponin-based Matrix-M (7, 8), are approved for use in human adults.

Ethical and safety risks warrant a careful evaluation of novel vaccines in the pediatric population. Generally, vaccine testing is performed in an age de-escalation manner, starting with adults, followed by adolescents, with children and infants being last (9). The BNT162b2 mRNA vaccine is now approved for use in adolescents 12 years and older, with trials for children ages 6 to 12 already under way (NCT04816643). The Moderna mRNA-1273 vaccine is close to approval for use in children 12 years and older. Moderna and Pfizer both have initiated clinical trials that will include infants as young as 6 months (NCT04796896 and NCT04816643, respectively). The important epidemiologic impact of pediatric SARS-CoV-2 vaccination lies in limiting transmission and ease of implementation via the global pediatric vaccine schedule.

Early in the pandemic, vaccines to prevent SARS-CoV-2 infection in children were not a priority because of apparent low infection and disease rates. In most children, SARS-CoV-2 infection causes only relatively mild disease. Nonetheless, some children develop severe symptoms, such as the multisystem inflammatory syndrome, requiring hospitalization and sometimes leading to death (10–14). Severe complications of SARS-CoV-2 infection disproportionally affect children of ethnic and racial minorities, amplifying health disparities in pediatric care in the United States (15, 16). Although children may transmit less efficiently than adults, virus transmission by children, even when asymptomatic, is documented (17, 18). Therefore, children have the propensity to become a major viral reservoir if pediatric vaccination stalls. The latter potential is especially concerning because new SARS-CoV-2 variants with increased transmission rates are emerging and SARS-CoV-2 has become more likely to persist on the population level. Although data are scarce, increased pediatric infection rates that appeared to coincide with the emergence of new variants have been reported (19, 20). There is ample precedence for the beneficial impact of pediatric vaccination.

Children have suffered from the SARS-CoV-2 pandemic in many additional ways. We will not know the exact burden of the loss of education due to closure of schools and virtual learning until years from now. School closure meant that many children were deprived of social interactions, a food source, and a safe place (21–23). There has been a dramatic rise in depression and anxiety in children, and the long-term mental health burden of these diverse factors is difficult to assess. School closure also translated to loss of work for many parents, especially women. A pediatric SARS-CoV-2 vaccine could mitigate at least some of these societal costs.

These combined data emphasize the importance of getting children vaccinated for SARS-CoV-2 (24–27). However, vaccine-elicited immunity differs between adults and infants [reviewed in (28)], with impaired responses to carbohydrate antigen vaccines (29) but higher magnitude humoral immunity to subunit-based vaccines (e.g., hepatitis B) (30) early in life. Thus, the evaluation of SARS-CoV-2 vaccine immunogenicity in infants is critical. Toward this goal, the present study was designed to evaluate the safety and immunogenicity of stabilized prefusion SARS-CoV-2 spike (S) vaccines delivered as lipid nanoparticle-encapsulated mRNA or an adjuvanted subunit protein in a relevant animal model. Nonhuman primates (NHP), including rhesus macaques (RMs), are an important model for SARS-CoV-2 studies given the similarities to humans in pathogenesis and host immune responses (31). Results from adult NHP studies of human SARS-CoV-2 vaccine candidates (32–35) strongly correlate with clinical trial outcomes, and infant NHP models of other infectious diseases closely mirror responses in human infants (36, 37). Data by our group support that infant vaccine-elicited Ab against HIV envelope (Env) can be of higher magnitude than those elicited by adults (38). We also demonstrated that infant RMs generate high-magnitude Ab responses to HIV Env protein vaccination (39) and that peripheral and mucosal B cell responses can be boosted later in life (40).

The primary goal of the current study was to inform human pediatric trials and aid in fast-tracking pediatric SARS-CoV-2 vaccines for inclusion in the early-life vaccine schedule by providing proof of concept that the two SARS-CoV-2 vaccines are safe and immunogenic in infant RMs. A secondary goal of our study was to assess the durability of vaccine-induced immune responses as data are lacking on this. Despite some caveats inherent to comparisons between studies with differences in study design, the immune responses in infant RMs in response to the two SARS-CoV-2 vaccines tested appear to be within the range of vaccine-induced immune responses reported for adult RMs (32, 41) and humans (2, 42, 43).

## RESULTS

### Study design and vaccine safety

**Fig. 1.**
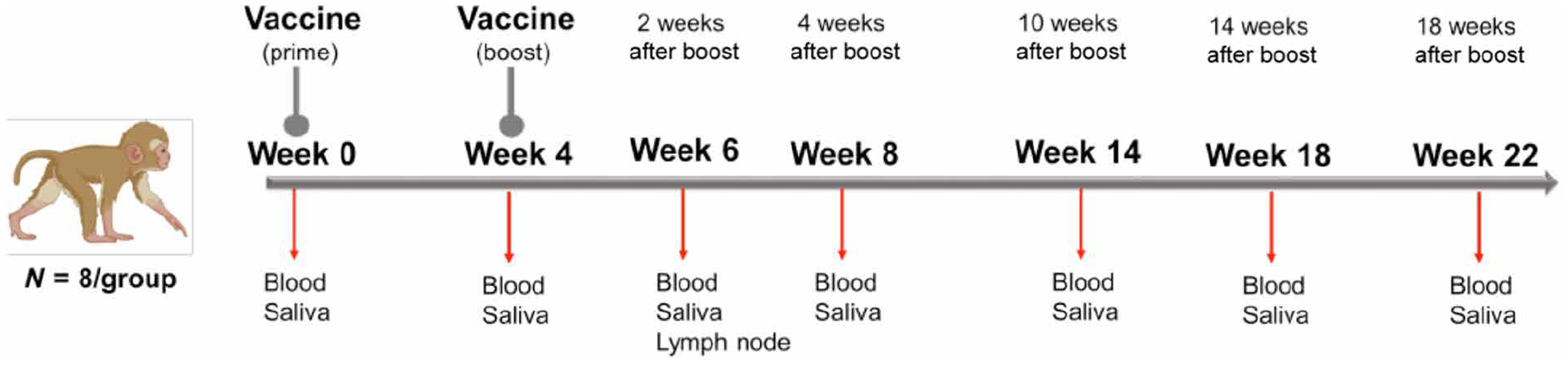
Study design: Evaluation of immunogenicity of two SARS-CoV-2 vaccines in infant RMs. Infant RMs (median age of 2.2 months at study initiation) were immunized at 0 and 4 weeks with either 30 μg of mRNA-encoding S-2P (Vaccine Research Center, NIH) in lipid nanoparticles (mRNA-LNP) or 15 μg of S-2P protein formulated with 3M-052 adjuvant, a TLR7/8 agonist, as a stable emulsion (3M-052-SE). Each group consisted of eight animals. Blood and saliva samples were collected at weeks 0, 4, 6, 8, 14, 18, and 22, and LN biopsies were obtained at week 6.

Infant RMs at a median age of 2.2 months, corresponding to 9-month-old human infants (44), were immunized at weeks 0 and 4 with 30 μg of mRNA encoding stabilized prefusion SARS-CoV-2 S-2P protein in lipid nanoparticles (mRNA-LNP; *n* = 8) or with 15 μg of S-2P mixed with 3M-052-SE, a Toll-like receptor 7/8 (TLR7/8) agonist in a stable emulsion (Protein+3M-052-SE; *n* = 8) (Fig. 1 and Table 1). Blood and saliva were collected before vaccination (week 0); 4 weeks after the first dose of the vaccine (week 4); 2 weeks after the vaccine boost (week 6); and at weeks 8, 14, 18, and 22. Lymph nodes (LNs) were sampled at week 6 (Fig. 1).

Animals were monitored daily for adverse events. No local injection site or systemic adverse reactions were observed. Animals had normal blood metrics (table S1). The animals gained weight consistent with adequate fluid and nutrition uptake and with normal infant growth (fig. S1) (45). A major safety concern in the implementation of a pediatric SARS-CoV-2 vaccine stems from adverse events observed with an earlier respiratory syncytial virus vaccine that were partially attributed to T helper 2 (T_H_2)–biased responses (46, 47). We therefore tested plasma samples before or after immunization for changes in T_H_2 [interleukin-4 (IL-4) and IL-13] or T_H_1 [IL-2 and interferon-γ (IFN-γ)] cytokines (fig. S2). Animal RM 3 of the protein group (Table 1) tested positive for all cytokines at both time points; cytokines were also detected in the mRNA-LNP recipient RM 11 at week 0 but fell below the limit of quantification by week 6. In general, neither vaccine appeared to induce systemic T_H_2 or T_H_1 responses (fig. S2), further supporting a good safety profile.

### Infant plasma and salivary Ab responses to SARS-CoV-2 vaccination

**Fig. 2.**
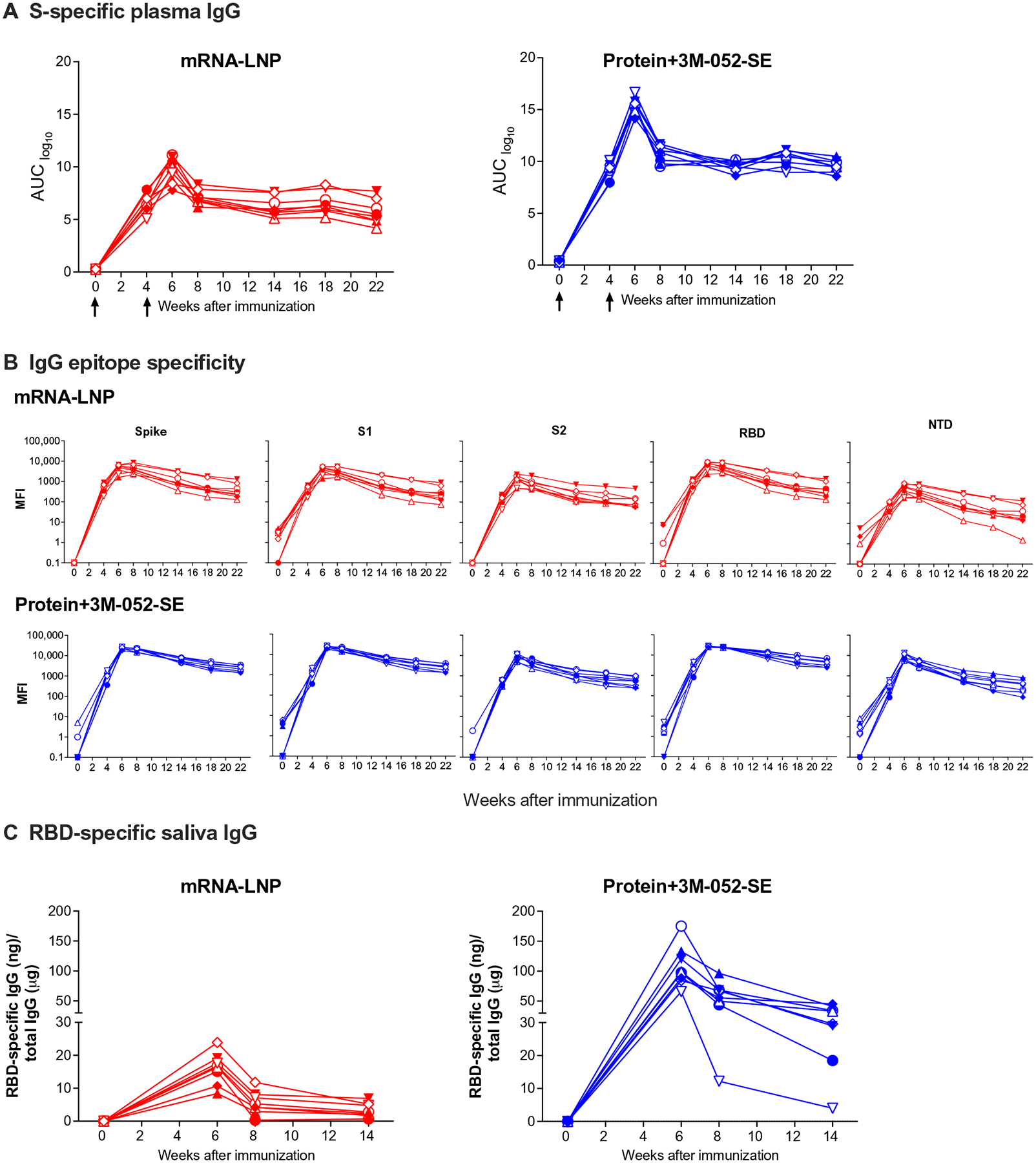
SARS-CoV-2 vaccine–elicited binding Ab responses in infant RMs. Plasma and saliva were collected before vaccination (week 0); at week 4 before the second dose; at 2 weeks after second dose (week 6); and at weeks 8, 14, 18, and 22 from infant RMs vaccinated with 30 μg of mRNA-encoding S-2P S protein in LNP (*n* = 8; red) or with 15 μg of prefusion SARS-CoV-2 S-2P S protein formulated with 3M-052 adjuvant (*n* = 8; blue). (**A**) S-2P protein–specific Ab responses were measured by ELISA. Serial dilutions of plasma starting at 1:40 were assayed for IgG binding to SARS-CoV-2 S. Data are reported as log_10_ AUC values. (**B**) Ab epitope specificity measured by binding antibody multiplex assay (BAMA). Plasma was diluted 1:10,000 to measure binding to different domains of the S protein, including the full-length S protein, S1, RBD, NTD, and S2. Binding Ab responses are reported as log_10_-transformed MFI after subtraction of background values. (**C**) Salivary RBD-specific IgG was measured by BAMA using serial dilutions of saliva, and responses are reported as RBD-specific IgG (nanograms)/total IgG (micrograms). Different symbols represent individual animals (Table 1). Arrows in (A) indicate times of immunizations.

Plasma immunoglobulin G (IgG) binding to the S-2P protein was observed after the vaccine prime for both mRNA-LNP and Protein+3M-052-SE vaccines and increased after the second immunization (Fig. 2). Although Ab levels dropped by week 8, there was only a slight decline of S-2P–specific IgG from week 8 to week 22 for mRNA-LNP [median area under the curve (AUC) and 95% confidence intervals: 7.0 (6.2, 8.3) to 5.4 (4.2, 7.7)] or Protein+3M-052-SE [11.0 (9.5, 11.7) to 9.4 (8.6, 10.5)] vaccinees (Fig. 2A). Longitudinal S-specific plasma IgG data reported as median effective concentration (EC_50_) or endpoint dilution titers followed a similar trend (fig. S3). Because the D614G virus variant had become dominant in the United States at the study initiation, we confirmed that IgG binding to S-2P D614G was similar to that of D614 at weeks 6 and 14 (fig. S4). Plasma IgG Abs were directed against multiple S domains and persisted throughout the study, with robust binding to S regions 1 and 2 (S1 and S2), receptor binding domain (RBD), and N-terminal domain (NTD) (Fig. 2B). RBD-specific salivary IgG from mRNA-LNP recipients peaked at a median of 16.6 ng of RBD-specific IgG per μg of total IgG at week 6 (Fig. 2C). In the Protein+3M-052-SE group, median salivary RBD-specific IgG peaked at IgG (98.2 ng/μg) after the second vaccination and remained detectable throughout the study (Fig. 2C). Saliva RBD-specific IgA responses were much lower (fig. S5). S-specific IgM and IgA in plasma were low or undetectable (fig. S5). In the mRNA-LNP group, IgM increased through week 14, but the absorbance readings for IgM were considered low, measured at a 1:10 sample dilution (fig. S5).

### Function of vaccine-induced plasma Abs

**Fig. 3.**
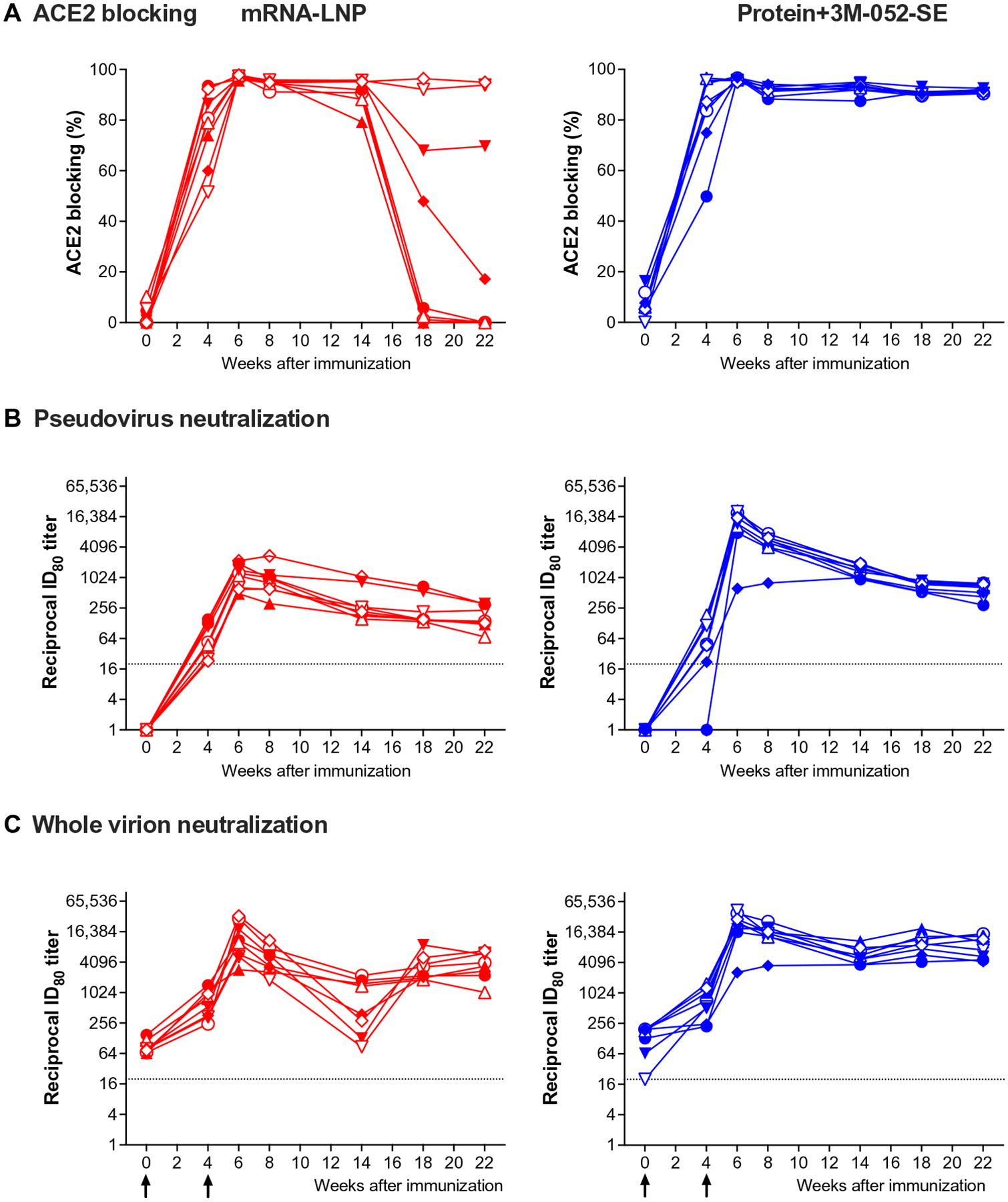
SARS-CoV-2 vaccine–elicited functional Ab responses in infant RMs. (**A**) The capacity of plasma Abs to mediate blocking of the RBD-ACE2 interaction was measured with an ELISA-based ACE2 blocking assay at 1:10 plasma dilution. Data are reported as ACE2 blocking (%). (**B** and **C**) Neutralization capacity was measured using S D614G-pseudotyped viruses in 293 T/ACE2 cells (B) and whole-virus (D614G) assay with Vero E6 cells (C); results are expressed as reciprocal 80% inhibitory dilution (ID_80_). Gray dotted lines represent detection cutoff. Different symbols represent individual animals (Table 1). Longitudinal data for each animal in the mRNA-LNP (red) or Protein+3M-052-SE group (blue) are represented by separate lines. Arrows in (C) indicate times of immunizations.

We next evaluated the capacity of plasma Abs to block entry of SARS-CoV-2 into human cells using a RBD-angiotensin converting enzyme 2 (ACE2) blocking assay at 1:10 and 1:40 plasma dilutions. Abs elicited by the mRNA-LNP vaccine completely blocked RBD-ACE2 interaction at week 6 at 1:10 and 1:40 dilutions. Greater than 80% blocking was achieved at 1:10 dilution until week 14, dropping for some animals after week 18 (Fig. 3A). The Protein+3M-052-SE vaccine induced Abs that mediated 100% RBD-ACE2 blocking after the second immunization (Fig. 3A). At a 1:40 plasma dilution, the RBD-ACE2 blocking function decreased over time but was still detectable at week 22 (fig. S6).

Neutralizing Abs, measured by a pseudovirus assay, were detected in all mRNA-LNP–vaccinated animals and in seven of eight animals of the Protein+3M-052-SE group after the first vaccination and further increased after the boost. Peak median inhibitory dilution 80 (ID_80_) titers were observed at week 6 and reached 1179 in mRNA-LNP and 13,687 in Protein+3M-052-SE animals (Fig. 3B). Similar to S-specific plasma IgG levels and consistent with Ab kinetics after vaccination, median ID_80_ neutralization titers decreased after the peak response. Neutralization titers appeared to stabilize around week 18, and median ID_80_ titers at week 22 remained 2.6-fold or 14.2-fold higher in the mRNA-LNP or Protein+3M-052-SE group, respectively, than after the first vaccination (week 4) (Fig. 3B). Although definite immune correlates of protection after vaccination still need to be conclusively determined, we modeled a biphasic Ab decline for both vaccines to reach a putative protective neutralizing Ab titer, estimated at a reciprocal dilution of 100 (41, 48). Applying this assumption, mRNA-LNP vaccinees maintained a protective neutralizing Ab titer for about 46 weeks and protein vaccine recipients for about 74 weeks (fig. S7).

Neutralizing Ab kinetics in the whole-virus neutralization assay followed a similar trend (Fig. 3C). The animal (RM 1) of the Protein+3M-052-SE group that did not have detectable neutralizing Abs by week 4 in the pseudovirus neutralization assay also only had a slight increase in the ID_80_ neutralizing Ab titer using the live virus assay (week 0, 129; week 4, 223). Overall, ID_50_ and ID_80_ titers of both assays strongly correlated with each other (*r* = 0.644, *P* = 0.008 or *r* = 0.785, *P* = 0.0005, respectively; fig. S8). However, infant RMs already had low-titer neutralizing Abs at day 0 when we applied the whole-virion neutralization assay (Fig. 3C). Because infants could have passively acquired Abs from their mothers, we measured neutralizing Abs in the sera of their unvaccinated dams. Although detectable, neutralizing Ab titers in dams did not correlate to infant neutralization titers before (ID_80_: *r* = 0.011, *P* = 0.96) or after vaccination (ID_80_: *r* = −0.056, *P* = 0.83) (fig. S9), most likely because infant RMs were already 2 months old at study initiation and maternal Abs had waned.

### Vaccine-elicited B cell responses

**Fig. 4.**
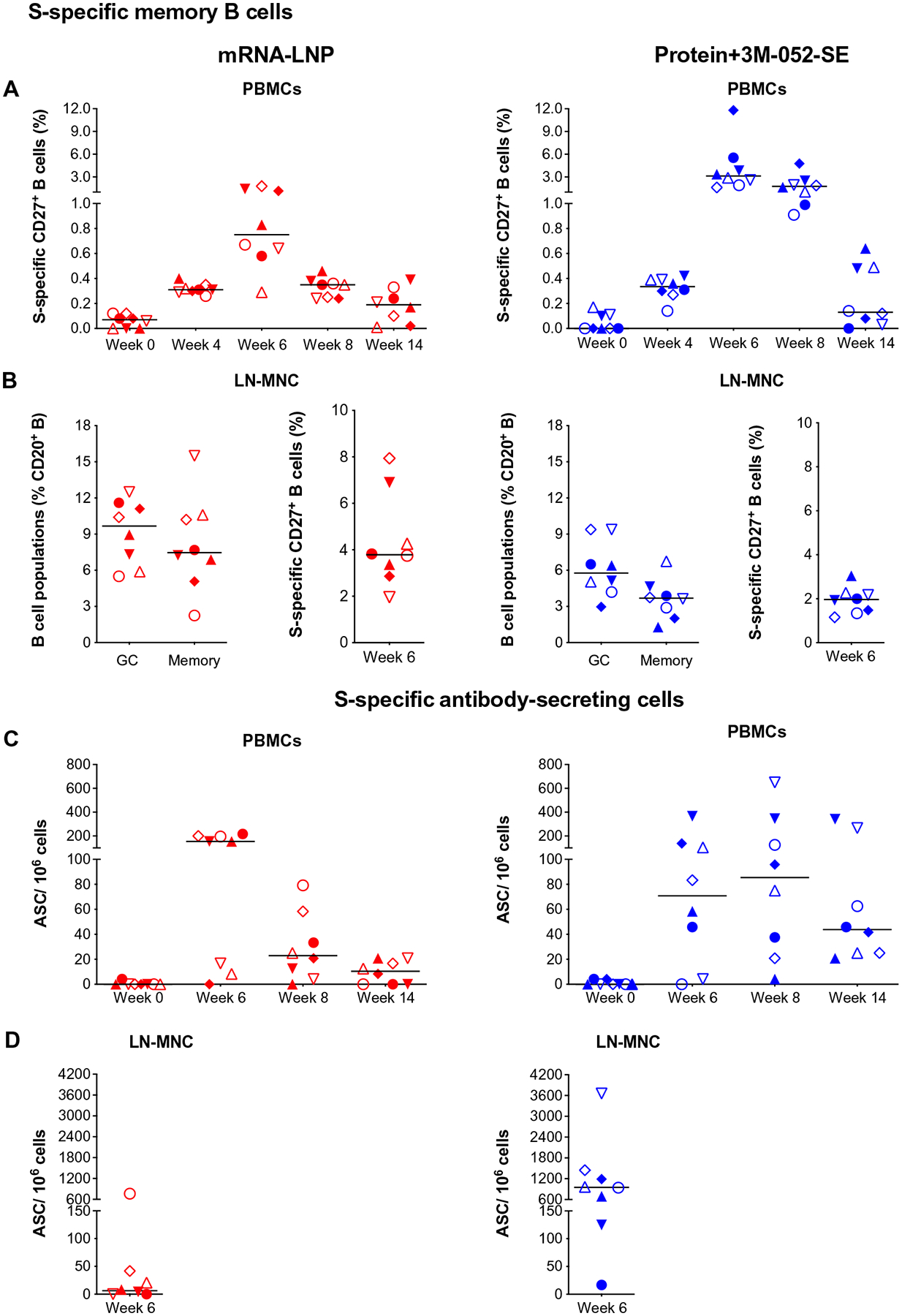
Characterization of S-specific B cell responses 2 weeks after boost. (**A**) CD20^+^CD27^+^ memory B cells that costained with fluorochrome-conjugated SARS-CoV-2 S protein in mRNA-LNP (red) or Protein+3M-052-SE (blue) vaccinees in blood. Frequencies are expressed as percent of total memory B cells. The gating strategy is provided in fig. S14. (**B**) In LNs, we determined total bcl-6^+^Ki-67^+^ GC B cells and CD27^+^ memory B cells as percent of total CD20^+^ B cells (left) (see fig. S15 for gating strategy) and also the percent of S-specific memory B cells (right). MNC, mononuclear cell. (**C**) ASC as measured by B cell ELISpot in PBMCs from mRNA-LNP or Protein+3M-052-SE vaccinees, whereas (**D**) is showing mRNA-LNP and Protein+3M-052-SE ASC responses, respectively, in LN at week 6. Different symbols represent individual animals (Table 1). Solid lines represent median values.

Consistent with the induction of plasma S-specific IgG, S-specific memory CD27^+^ B cells were detectable in the blood of both vaccine groups at week 4 and peaked at week 6 (median: 0.75% mRNA-LNP and 3.12% Protein+3M-052-SE). At week 14, median memory B cell frequencies were lower (0.19 or 0.13% in mRNA-LNP or Protein+3M-052-SE vaccinees, respectively) but did not differ from those at week 4 (Fig. 4A). In LNs, we measured total germinal center (GC) B cells and memory B cells and then determined the number of S-specific memory B cells (Fig. 4B). In both vaccine groups, robust memory B cell populations (median: 3.78% mRNA-LNP and 1.96% Protein–3M-052) were present (Fig. 4B). Induction of S-specific B cells was confirmed by assessing S-specific Ab-secreting cells (ASC). In the blood, peak responses were observed at week 6 in the mRNA-LNP group (median: 154 ASC per million cells) and at week 8 in the Protein+3M-052-SE group (median: 85 ASC per 10^6^ cells) (Fig. 4C). In draining LNs, mRNA-LNP and Protein+3M-052-SE vaccinees had median 6 or 952 ASC per 10^6^ cells, respectively (Fig. 4D).

**Fig. 5.**
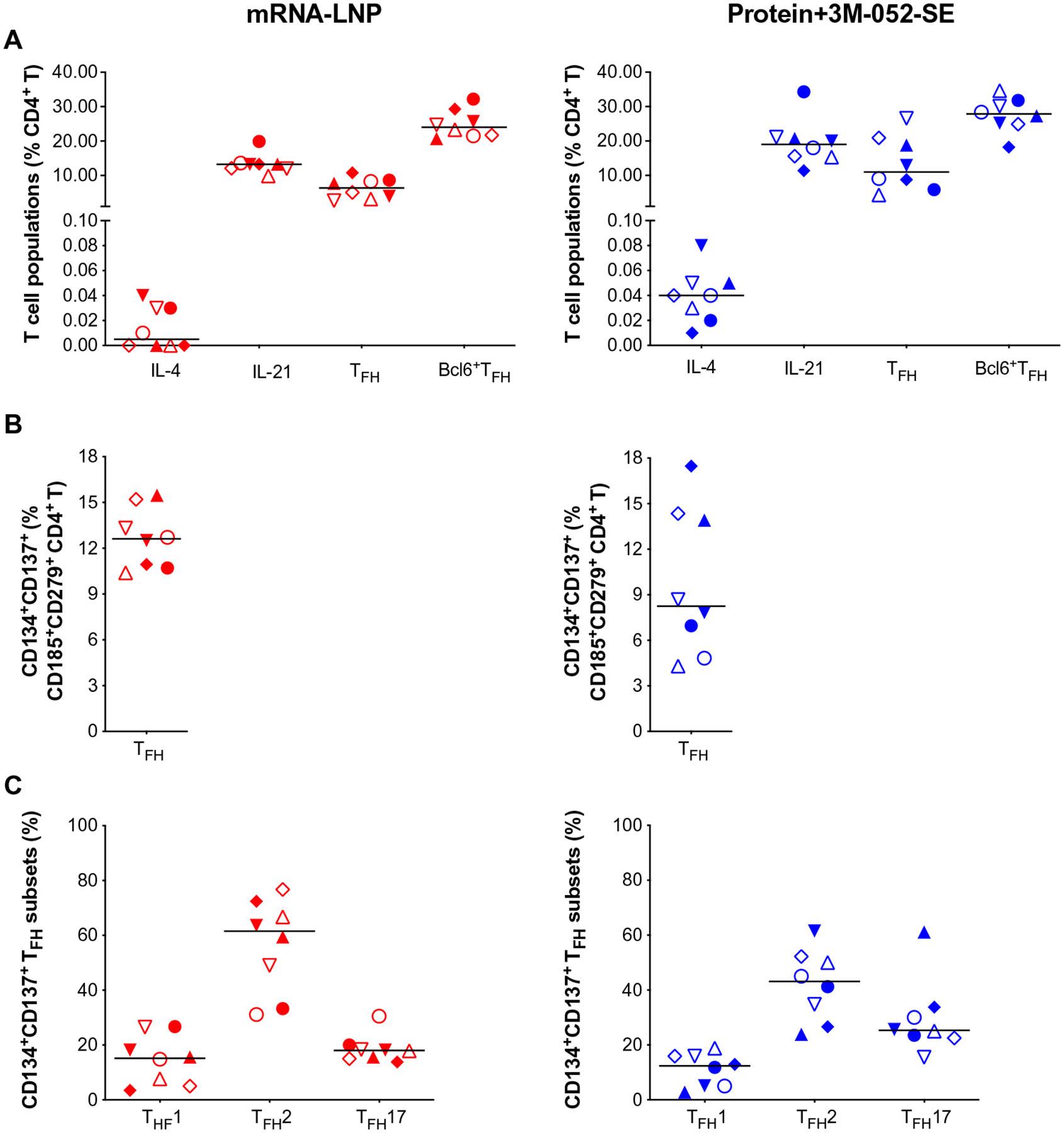
Immunophenotype of LN T cell population 2 weeks after boost. (**A**) CD4^+^ T cells positive for IL-4 or IL-21, T_FH_ markers CD279/PD-1 and CD185/CXCR5, or bcl6^+^ T_FH_ were measured and are represented as percent of total LN CD4^+^ T cells. The gating strategy for these panels is provided in fig. S15. (**B**) SEB-activated T_FH_ frequencies assessed by the AIM assay: CXCR5^+^CD185^+^ cells that coexpressed CD134 and CD137 for Protein+3M-052-SE and mRNA-LNP, respectively. (**C**) The frequency of CXCR5^+^CD185^+^CD134^+^CD137^+^CXCR3^+^CD196^−^ T_FH_1, CXCR3^−^CCR6^−^ T_FH_2, and CXCR3^−^ CD196^+^ T_FH_17 cells is shown. The gating strategy for these populations is given in fig. S17. Red symbols, mRNA-LNP; blue symbols, Protein+3M-052-SE. Different symbols represent individual animals (Table 1). Solid lines define the median.

In addition, we analyzed LN samples for canonical T follicular helper cells (T_FH_) (CD185/CXCR5^+^CD279/PD-1^+^) and for IL-4– or IL-21–producing CD4^+^ T cells that support GC B cell differentiation (Fig. 5A). Staphylococcal enterotoxin B (SEB)–activated T_FH_ (CD185^+^ CD279^+^CD134^+^CD137^+^) (Fig. 5B) (49) were categorized into T_FH_1, T_FH_2, and T_FH_17 subsets on the basis of expression of CXCR3/CD183 and/or CCR6/CD196 (Fig. 5C) (50). We found no correlations between bcl6^+^Ki67^+^ GC B cells or bcl6^+^T_FH_ (fig. S10, A and B), potentially because we assessed these cell populations in an antigen-nonspecific manner. However, CD4^+^IL-21^+^ cells correlated with pseudovirus neutralizing Ab ID_50_ at week 6 (*r* = 0.749, *P* = 0.001) (fig. S10C).

### S protein–specific T cell responses

**Fig. 6.**
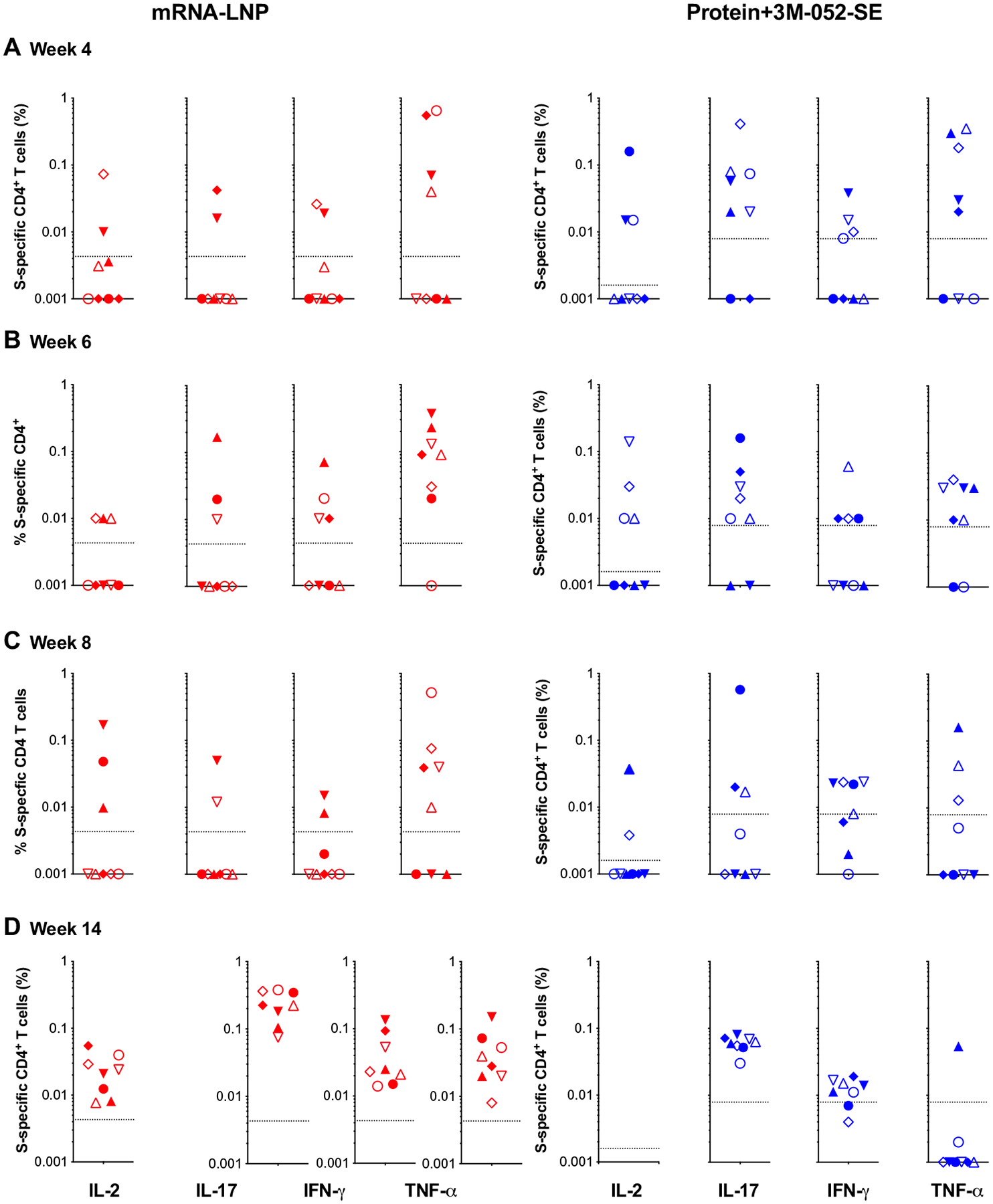
S-specific CD4^+^ T cell responses in SARS-CoV-2 immunized infant macaques. Intracellular cytokine staining for IL-2, IL-17, IFN-γ, and TNF-α [indicated at the *x* axis in (D)] was performed on PBMCs at weeks 4 (**A**), 6 (**B**), 8 (**C**), and 14 (**D**) to assess T cell responses to a peptide pool encompassing the entire SARS-CoV-2 S protein (see fig. S16 for gating strategy). Responses detected in mRNA-LNP recipients are displayed in red, and cytokine responses from Protein+3M-052 and SE vaccinees are in blue. The dashed lines represent week 0 values plus 2 SDs and define the cutoff for positive cytokine responses. Different symbols represent individual animals (Table 1).

In some animals, S-specific CD4^+^ T cell responses in peripheral blood mononuclear cells (PBMCs) were detected as early as week 4, with all animals producing at least a single cytokine by week 6 (Fig. 6; see fig. S16 for gating strategies). At week 14, CD4^+^ T cells of mRNA-LNP–vaccinated animals produced IL-2, IFN-γ, IL-17, and tumor necrosis factor–α (TNF-α) responses (Fig. 6D), whereas IL-17 and IFN-γ CD4^+^ T cell responses dominated in Protein+3M-052-SE vaccinees (Fig. 6D). Multifunctional CD4^+^ T cells coproduced IL-17 and IFN-γ (fig. S11), suggesting a T_H_1/T_H_17-biased response. Although we did not measure IL-4 production and limited cell numbers—typical for pediatric samples—prevented us from repeating the assay, it should be reiterated that neither of the vaccines caused a rise in systemic T_H_2 cytokines (fig. S2). S-specific peripheral blood CD8^+^ T cell responses appeared less robust than CD4^+^ T cell responses and only single-cytokine positive CD8^+^ T cells were elicited, but all animals produced at least a single cytokine in response to antigen stimulation (fig. S12). In LNs, six of eight and eight of eight animals in the mRNA-LNP or Protein+3M-052-SE group had S-specific CD4^+^ T cell responses, respectively. In addition, eight of eight mRNA-LNP vaccinees and seven of eight Protein+3M-052-SE vaccinees mounted S-specific CD8^+^ T cell responses in LN at week 6 (fig. S13).

Overall, these data suggested that infant RMs can mount robust Ab, including high-titer neutralizing and ACE2-blocking Abs, and T cell responses to SARS-CoV-2 vaccines. Vaccine-induced immune responses persisted for 22 weeks or 18 weeks after the first and second vaccine doses, respectively, results consistent with vaccine-induced memory.

## DISCUSSION

Several SARS-CoV-2 vaccines have demonstrated safety, immunogenicity, and protection in animal studies (8, 32, 51, 52) and in clinical trials with human adults (1, 5, 6) and subsequently received authorization for emergency use in persons greater than 12 years old. Considering the enormous health and socioeconomic impact of the SARS-CoV-2 pandemic on all age groups, clinical trials to test the safety of SARS-CoV-2 vaccines in adolescents and children have been initiated. The Pfizer-BioNTech mRNA vaccine is already approved for adolescents 12 years and older, Moderna’s vaccine (NCT04796896) has recently been reported to be 96% effective in adolescents ages 12 to 17, and Novavax (NCT04611802) has an ongoing trial to test its vaccine in this age group. However, data regarding the safety and immunogenicity of SARS-CoV-2 vaccines in young infants are still lacking.

We reasoned that validating safety and immunogenicity of SARS-CoV-2 vaccines in infant RMs would provide beneficial information supporting the initiation of pediatric vaccine trials down to 6 months of age. Here, we present preclinical data demonstrating that infant RMs develop strong, durable humoral, and cellular responses in the absence of adverse events after vaccination with a preclinical version of the Moderna mRNA-1273 vaccine or with stabilized prefusion S-2P SARS-CoV-2 protein mixed with 3M-052-SE, a TLR7/8 agonist in squalene emulsion. We selected the mRNA-LNP vaccine expressing S-2P because at the initiation of the study, mRNA vaccines were quickly progressing through phase 3 clinical studies toward approval for human use, with emergency use authorization approval in the United States in late 2020. We therefore considered this to be the class of vaccines most likely to be among the first vaccines to eventually progress for testing in the pediatric population. The 3M-052-SE adjuvant was chosen for the S-2P protein vaccine because this adjuvant has proven effective in eliciting high-magnitude Ab responses to other vaccines in infant RMs (39, 53).

We assessed infant vaccine-induced immune responses over 22 weeks, analogous to adult clinical trials (1, 2). Both vaccines elicited plasma Abs dominated by IgG and recognized all S protein domains. Binding Abs persisted throughout the study in all animals of both groups. RBD-specific IgG was also detected in saliva, especially in animals of the protein vaccine group, and remained detectable throughout the study, similar to what has been observed after human natural infection (54). Moreover, seven of eight Protein+3M-052-SE–vaccinated animals had RBD-specific IgA after the second immunization. The overall low induction of S-2P– and RBD-specific IgA by vaccination might be attributed to the delayed development of mucosal IgA compared with IgG responses in human and rhesus infants (55–57). Vaccine-induced neutralizing Ab kinetics paralleled those observed for plasma binding Abs and persisted for the duration of the study. At week 22, 18 weeks after the second dose, median ID_50_ titers in the pseudovirus assay still exceeded 10^3^ for the protein vaccine group and 10^2^ for the mRNA group.

The immune correlates of protection against SARS-CoV-2 infection and disease still need to be conclusively determined (58, 59). To address this question, McMahan *et al*. (48) adoptively transferred plasma IgG from adult SARS-CoV-2 convalescent RMs and determined that an S protein–specific reciprocal endpoint dilution ELISA titer of 400 and pseudovirus neutralizing Ab (ID_50_) titers of about 50 can protect adult RMs against combined intratracheal and intranasal SARS-CoV-2 challenge (48). Applying these criteria to our results, we hypothesized that pediatric vaccination could provide protection against SARS-CoV-2 infection. However, we purposely decided to delay the challenge of our animals until 1 year after vaccination to better determine the long-term persistence of vaccine-induced neutralizing Abs, which is especially important to a pediatric vaccine for a disease that affects all age groups. These data will be reported in a follow-up study. Furthermore, the drop in ACE2-blocking function in some animals of the mRNA-LNP group raises the possibility that some of our vaccinated animals might develop disease, whereas others would be protected. Such an outcome not only would allow us to define immune correlates of protection, including the protective titer of neutralizing Abs against infection and from severe disease, but also enable us to assess the contribution of other vaccine-induced immune responses (e.g., T cell responses) to protection.

Early-life immunity is associated with T_H_2-biased T cell responses (60), and T_H_2 responses have been linked to vaccine-associated enhanced respiratory disease in the context of protein or inactivated virus vaccines (6, 61, 62). We observed a T_H_1/T_H_17-skewed cytokine profile in circulating S-specific T cells. Our findings of peak S-specific T cell responses 10 weeks after boost are not unexpected. T cell reactivity in convalescent patients with coronavirus disease 2019 (COVID-19) has augmented frequency and potency 100 days after recovery, even as S-specific Ab waned (63). “Exposed asymptomatic” patients (~93%) have SARS-CoV-2–specific memory T cell subsets in the absence of sero-conversion (64). Higher T_FH_2 frequencies compared with T_FH_1 do not necessarily imply a T_FH_2 bias because T_FH_17 were also detected, and T_FH_ were not assessed for antigen specificity. We found no evidence of systemic T_H_2 cytokines before or after immunization in either vaccine group, corroborating previous findings in adult macaques (32) and adult humans (42) that also demonstrated low-level or absent T_H_2-mediated responses. IL-4– and IL-21–producing T_FH_ are required for the induction of GC B cells in LNs (65), indispensable for B cell and Ab affinity maturation, and GC reactions are important for positive outcomes in patients with COVID-19 (65). The persistence of Ab was paralleled by sustained S-specific B cell responses. The induction and persistence of S-specific B cell clones described here may be key to protection against reinfection (66). In the current study, S-specific CD8^+^ T cell responses were lower compared with CD4^+^ T cell responses in the same animals. Because the CD8^+^ T cell response plays an important role in the control of virus replication, future studies need to determine the contribution of vaccine-induced cytotoxic T cell responses to protection against SARS-CoV-2 infection.

Overall, the adjuvanted protein vaccine seemed to elicit stronger immune responses compared with the mRNA vaccine. We can only speculate why the vaccine-induced immune responses were different between the Protein+3M-052-SE and the mRNA-LNP regimens. First, we do not know the exact amount of protein expressed by the mRNA-LNP vaccine in vivo. Second, we and others have previously demonstrated that the TLR7/8-based adjuvant 3M-052 is highly effective in enhancing Ab responses in the RM model (39, 53). TLR7/8 agonists, in contrast to most other TLR agonists, promote potent IL-12 responses by antigen-presenting cells (67–69) that, in neonates and young infants, are normally biased toward IL-23 production (70). Therefore, the priming of T_H_ cells and Ab responses is assumed to be improved by the inclusion of the 3M-052-SE adjuvant. Nonetheless, the data do not imply that the mRNA-LNP vaccine was less successful in inducing immune responses compared with responses observed in human clinical trials or in adult NHP. S-specific T and B cell responses to the mRNA-LNP vaccine persisted throughout the study period.

It is difficult to directly compare the results of the current study with published data from human clinical trials or from adult NHP studies. We are not aware of published data using the Protein+3M-052-SE vaccine for SARS-CoV-2 in NHP. Results from human and NHP studies with the mRNA-1273 vaccine are listed in Table 2 (2, 32, 41–43). It should be noted that the various studies differed in their design. Keeping this caveat in mind, the magnitude, quality, and durability of the mRNA-LNP vaccine–induced responses in infant macaques appear to be within the range of those observed in adult NHP and human studies.

We demonstrated that infant RMs mount strong and durable responses to mRNA-LNP and protein-based SARS-CoV-2 vaccines that were comparable to adults without adverse reactions. These promising results endorse clinical translation of SARS-CoV-2 vaccines to early-life populations. Even when most adults and adolescents have been vaccinated and herd immunity is achieved, globally about 140 million infants are born each year (71), and they will be naïve and susceptible to SARS-CoV-2 once passively transferred maternal Abs wane. Being cautiously optimistic, a multivalent SARS-CoV-2 vaccine effective against major circulating viral variants appears feasible and could become part of the standard pediatric vaccine program.

## MATERIALS AND METHODS

### Study design

The objective of this study was to provide proof of concept that young infants (<1 year) could mount functional and durable neutralizing Ab responses and T cell responses to SARS-CoV-2 vaccination. Considering the extent of the SARS-CoV-2 pandemic, the rapid emergence of new viral variants with increased transmission rates with yet unknown impacts on pediatric infections, our goal was to inform human SARS-CoV-2 vaccine age de-escalation studies to fast-track vaccine implementation in the pediatric population. Using the highly relevant infant RM model, we tested the safety and immunogenicity of a preclinical version of the mRNA 1273 SARS-CoV-2 vaccine (*n* = 8) and a stabilized prefusion SARS-CoV-2 S-2P S protein vaccine mixed with the 3M-052 adjuvant in stable emulsion (*n* = 8) (Fig. 1 and Table 1). S-specific cellular responses and Ab responses were monitored for 22 weeks.

### Animals

Infant male (*n* = 8) and female (*n* = 8) RMs (*Macaca mulatta*) of Indian origin from the California National Primate Research Center (CNPRC, Davis, CA) breeding colony (negative for type D retrovirus, simian immunodeficiency virus, simian lymphocyte tropic virus type 1, and SARS-CoV-2) were enrolled at a median age of 2.2 months and randomly assigned into two groups (Table 1). Infants were housed with their dams until about 6 months of age and then weaned and pair-housed. Animal care was in compliance with the *Guide for Care and Use of Laboratory Animals* by the Institute for Laboratory Animal Research. Animal procedures were approved by the University of California Davis Institutional Animal Care and Use Committee before study initiation. All procedures were performed under anesthesia (ketamine, 10 mg/kg body weight, intramuscularly). Blood, saliva, and LN were collected and processed as described (72).

### Preparation of the SARS-CoV2-S protein

The CoV-2 S (73) (2019-nCoV) protein was transiently expressed in FreeStyle human embryonic kidney (HEK) 293 cells (Thermo Fisher Scientific) by first diluting the plasmid in Opti-MEM I (Gibco) medium; 0.8 mg of plasmid in 25 ml of Opti-MEM I for each liter of cells was transfected and sterile-filtered using a 0.22-μm filter. 293fectin (1 ml; Gibco) was diluted in 25 ml of Opti-MEM I and allowed to incubate at room temperature (RT) for 5 ± 1 min. The plasmid and the 293fectin mixtures were combined and swirled to mix and then incubated at RT for 25 ± 5 min. During the incubation, the FreeStyle HEK293 cells were diluted to 1.25 × 10^6^ cells/ml in a 2-liter Corning flask. At the end of the 25 ± 5-min incubation of the 293fectin and plasmid mixtures, 50 ml of the transfection mixture was added to each flask of diluted cells with gentle agitation of the flask during addition. The cell and transfection mixture was then split between two 2-liter Corning flasks for a final volume of about 500 ml each and placed on a platform shaker in a humidified incubator at 37°C with 8% CO_2_. The shaker speed was set to 120 rpm, and the flasks were allowed to incubate for 6 days. At the end of the 6-day incubation, the flasks containing the transfected cells were removed from the incubator, and the contents of the flasks were aseptically transferred to 500-ml Corning centrifuge tubes. The supernatant was clarified by centrifugation and subsequently filtered using a 0.8-μm filter bottle system. Keeping the clarified and filtered supernatant on ice, the supernatant was concentrated using a Sartorius Vivaflow 200 30-kDa tangential flow filtration system. In preparation for the purification of the CoV-2 S protein, 4 ml of Strep-Tactin resin (IBA Lifesciences) was placed in a conical tube. The concentrated supernatant was transferred to the conical tube containing the Strep-Tactin resin and allowed to bind with gentle agitation. The supernatant and resin were then transferred to a polypropylene column for gravity-flow chromatography (Bio-Rad). Once the resin had settled in the column, the resin was washed and the protein eluted following instructions supplied by the vendor (IBA Lifesciences). The eluate was buffer-exchanged into 2 mM tris and 200 mM sodium chloride storage buffer using Millipore centrifugal filters. The resulting material was lastly filtered through a 0.2-μm syringe filter (Pall) and stored in storage buffer. Additional purification by size exclusion chromatography over a Superose 6 column was performed to increase purity.

### Vaccines

The SARS-CoV-2 stabilized prefusion S (S-2P) mRNA-LNP vaccine was provided by Moderna Inc., and the Vaccine Research Center [National Institutes of Health (NIH)] provided the S-2P protein. The vaccine regimen and specimen collection are outlined in Fig. 1. In humans, the dose of the Moderna mRNA-1273 vaccine is 100 μg. However, even a 25-μg dose has proven immunogenic and was associated with fewer side effects compared with the 100 μg in older adults (42). In adult RMs, a 10-μg dose regimen induced significantly lower neutralizing Ab responses than the 100-μg vaccine (32). Thus, balancing immunogenicity and safety, we decided to immunize infant RMs in the mRNA-LNP vaccine group intramuscularly at weeks 0 (quadriceps) and 4 (biceps) with 30 μg of mRNA-encoding S-P2 protein in lipid nanoparticles (mRNA-LNP), administered in 0.1 ml of phosphate-buffered saline (PBS). Note that the vaccine was stored at −80°C until just before the immunization. Infant RMs in the protein vaccine group were injected intramuscularly with15 μg of S-2P protein mixed with 3M-052-SE, an adjuvant formulation consisting of 10 μg of the synthetic TLR7/8 agonist 3M-052 in a 2% (v/v) squalene-in-water emulsion (Protein+3M-052-SE) in 0.5 ml divided across the left and right quadriceps (week 0) or biceps (week 4).

### Plasma IgG ELISA

IgG binding to the stabilized SARS-CoV-2 S protein S-2P with or without the D614G mutation was measured in plasma using ELISA as previously described (74). Three hundred eighty-four–well plates were coated overnight with S protein (2 μg/ml) produced by the Protein Production Facility (PPF) at the Duke Human Vaccine Institute. Plates were then blocked with assay diluent (PBS containing 4% whey, 15% normal goat serum, and 0.5% Tween 20). Ten serial fourfold dilutions of plasma starting at 1:40 were added to the plates and incubated for 1 hour, followed by detection with a horse-radish peroxidase (HRP)–conjugated mouse anti-monkey IgG (SouthernBiotech). The plates were developed by using an 2,2′-azinobis(3-ethylbenzthiazolinesulfonic acid peroxidase substrate system (Colonial Scientific), and absorbance was read at 415 nm with a SpectraMax microplate reader (Molecular Devices). Results were calculated as AUC and EC_50_ values. AUC values were calculated using the trapezoidal rule. EC_50_ values were calculated by fitting a four-parameter logistic function using nonlinear regression. Pooled NHP convalescent serum to SARS-CoV-2 (BEI Resources, NR-52401) was used in all assays to ensure interassay reproducibility, but standard curves were not developed given the lack of an RM-specific IgG reagent of known concentration. IgM and IgA binding to S-2P was measured in a single 1:10 diluted plasma sample following the same ELISA protocol but using as detection Ab anti-human IgM HRP (Jackson ImmunoResearch) for IgM detection and 10F12-biotin (NHP Reagent Resource) with Streptavidin-HRP (Pierce) for IgA detection. Results are expressed as optical density at 450 nm (OD_450_).

### Measurement of salivary S-specific IgG and IgA

Saliva was collected with absorbent Merocel sponges (Beaver Visitec) by placing a sponge between the cheek and gum in the back of the mouth for 5 min. Secretions were eluted by centrifugation at 18,000*g* and 4°C after addition of 50 μl of PBS containing protease inhibitors (75), 1% Triton X-100, 1% bovine serum albumin (BSA), 0.05% azide, and 0.05% Tween 20 to sponges. A customized binding antibody multiplex assay (BAMA) was used to measure IgG or IgA Abs to SARS-CoV-2 recombinant RBD protein (provided by J. Wrammert, Emory University, Atlanta, GA) and S2 extracellular domain (Sino Biological no. 40590-V08B, Wayne, PA). Briefly, proteins were dialyzed in PBS and conjugated to Bio-Plex Pro carboxylated magnetic beads (Bio-Rad, Hercules, CA) using *N*-hydroxysulfosuccinimide and ethylcarbodiimide as described (76). Serial dilutions of standard and centrifuged salivary secretions in PBS containing 1% Triton X-100, 1% BSA, 0.05% azide, and 0.05% Tween 20 were mixed with RBD and S2 beads overnight at 1100 rpm and 4°C using a plate mixer. The IgG standard was a cocktail of anti-S1 RBD (GenScript, no. HC2001) and anti-S2 (Sino Biological, no. 40590-D001) humanized IgG monoclonal Abs. The standard for IgA assays was a pooled serum from infected RMs (77) that had been calibrated relative to the previously mentioned monoclonal Abs. The following day, beads were alternately washed using a Bio-Rad Bio-Plex wash station and treated for 30 min with biotinylated affinity-purified goat Ab (2 μg/ml) to human γ chain (SouthernBiotech Associates, Birmingham, AL) or clone IgA5–3B mouse anti-monkey IgA (Bio-Rad) followed by 1/400 avidin-phycoerythrin (PE; SouthernBiotech). A Bio-Rad Bio-Plex 200 and BioManager software were used to measure fluorescent intensity and construct standard curves for interpolation of Ab concentrations in test samples. Concentrations of Ab were normalized relative to the total IgG and IgA measured by ELISA as described (75) using plates coated with goat anti-monkey IgG or IgA and the secondary Abs above.

### Plasma S-specific IgG epitope mapping

SARS-CoV-2 antigens, including whole S (produced by PPF), S1 (Sino Biological, catalog no. 40591-V08H), S2 (Sino Biological, catalog no. 40590-V08B), RBD (Sino Biological, catalog no. 40592-V08H), and NTD (Sino Biological, catalog no. 40591-V49H) were conjugated to Magplex beads (Bio-Rad, Hercules, CA). The conjugated beads were incubated on filter plates (Millipore, Stafford, VA) for 30 min before plasma samples were added. Plasma samples were diluted in assay diluent [1% dry milk, 5% goat serum, and 0.05% Tween 20 in PBS (pH 7.4.)] at a 1:10,000-point dilution. Beads and diluted samples were incubated for 30 min with gentle rotation, and IgG binding was detected using a PE-conjugated mouse anti-monkey IgG (SouthernBiotech, Birmingham, Alabama) at 2 μg/ml. Plates were washed and acquired on a Bio-Plex 200 instrument (Bio-Rad, Hercules, CA), and IgG binding was reported as mean fluorescence intensity (MFI). To assess assay background, the MFIs of wells without sample (blank wells) were used, and nonspecific binding of the samples to unconjugated blank beads was evaluated.

### RBD-ACE2 blocking assay

Corning 384-well plates were coated with ACE2 protein (3.5 μg/ml; Sino Biological) 24 hours before conducting the experiment. The day of the assay, the plate was blocked with assay diluent (PBS containing 4% whey, 15% normal goat serum, and 0.5% Tween 20) for 1 hour at RT. Plasma was diluted 1:10, 1:40, or 1:60 using the assay diluent and incubated with HRP-RBD protein (1 μg/ml; GenScript) at 37°C for 1 hour. Plates were washed and the preincubated mixture of plasma and HRP-RBD protein was added in duplicate and incubated at RT for 1 hour. Then, plates were washed four times to remove unbound sample, and peroxidase substrate solution (SeraCare) was added for 4 min before stopping the reaction using stop solution (SeraCare). Signal was detected using a SpectraMax plate reader at OD_450_. The amount of signal detected in wells without sample (diluent only) was considered the maximal binding response, and the OD_450_ detected in the sample wells was transformed to percentage inhibition compared with maximum binding. Data presented are the average of two replicates when the coefficient of variation was less than 20%, and if different assays were performed, then median value was considered.

### Pseudovirus Ab neutralization assay

SARS-CoV-2 neutralization was assessed with S-pseudotyped viruses in 293 T/ACE2 cells as a function of reductions in luciferase (Luc) reporter activity. 293 T/ACE2 cells were provided by M. Farzan and H. Mu at Scripps Florida. Cells were maintained in Dulbecco’s modified Eagle’s medium containing 10% fetal bovine serum (FBS), 25 mM Hepes, gentamycin (50 μg/ml), and puromycin (3 μg/ml). An expression plasmid encoding codon-optimized full-length S of the Wuhan-1 strain (VRC7480) was provided by B.S.G. and K.S.C. at the Vaccine Research Center, NIH (USA). The D614G amino acid change was introduced into VRC7480 by site-directed mutagenesis using the QuikChange Lightning Site-Directed Mutagenesis Kit from Agilent Technologies (catalog no. 210518). The mutation was confirmed by full-length S gene sequencing. Pseudovirions were produced in HEK293 T/17 cells (American Type Culture Collection, catalog no. CRL-11268) by transfection using Fugene 6 (Promega, catalog no. E2692) and a combination of S plasmid, lentiviral backbone plasmid (pCMV ΔR8.2), and firefly Luc reporter gene plasmid (pHR’ CMV Luc) (78) in a 1:17:17 ratio. Transfections were allowed to proceed for 16 to 20 hours at 37°C. Medium was removed, monolayers were rinsed with growth medium, and 15 ml of fresh growth medium was added. Pseudovirus-containing culture medium was collected after an additional 2 days of incubation and was clarified of cells by low-speed centrifugation and 0.45-μm filtration and stored in aliquots at −80°C. Median tissure culture infectious dose assays were performed on thawed aliquots to determine the infectious dose for neutralization assays.

For neutralization, a pretitrated dose of pseudovirus was incubated with eight serial fivefold dilutions of serum samples in duplicate in a total volume of 150 μl for 1 hour at 37°C in 96-well flat-bottom poly-l-lysine–coated culture plates (Corning Biocoat). Cells were suspended using TrypLE Select Enzyme solution (Thermo Fisher Scientific) and immediately added to all wells (10,000 cells in 100 μl of growth medium per well). One set of eight control wells received cells and virus (virus control), and another set of eight wells received cells only (background control). After 66 to 72 hours of incubation, medium was removed by gentle aspiration and 30 μl of Promega 1X lysis buffer was added to all wells. After a 10-min incubation at RT, 100 μl of Bright-Glo Luc reagent was added to all wells. After 1 to 2 min, 110 μl of the cell lysate was transferred to a black/white plate (PerkinElmer). Luminescence was measured using a PerkinElmer Life Sciences, Model Victor2 luminometer. Neutralization titers are the serum dilution at which relative light units (RLUs) were reduced by either 50% (ID_50_) or 80% (ID_80_) compared with virus control wells after subtraction of background RLUs. Serum samples were heat-inactivated for 30 min at 56°C before assay.

### Whole-virus neutralization assay

Neutralization of SARS-CoV-2 nanoLUC carrying the D614G mutation was assessed as described by Hou *et al*. (79) with modifications. Briefly, under BSL-3 containment, serially diluted sera at eight dilutions were incubated for 1 hour with SARS-CoV-2 D614G nanoLUC virus at 5% CO_2_ and 37°C. After incubation, the virus/Ab mixtures were added in duplicate to black 96-well plates containing Vero E6 cells (2 × 10^4^ cells per well). Each plate contained virus-only (no serum) control wells. The plates were incubated for 24 hours at 37°C, 5% CO_2_, the cells lysed, and Luc activity measured with the Nano-Glo Luciferase Assay System (Promega). Neutralization activity was expressed as the dilution concentration at which the observed relative light units (RLUs) were reduced by 50% or 80% relative to virus-only control wells.

### Preparation of S-specific hook reagents for flow cytometry

To express the prefusion S ectodomain, a gene encoding residues 1 to 1208 of 2019-nCoV S (GenBank, MN908947) with proline substitutions at residues 986 and 987, a GSAS substitution at the furin cleavage site (residues 682 to 685), a C-terminal T4 fibritin trimerization motif, an HRV3C protease cleavage site, a Twin-Strep-Tag, and an 8XHisTag was synthesized and cloned into the mammalian expression vector pαH. To express the 2019-nCoV RBD-SD1, residues 319 to 591 of 2019-nCoV S were cloned upstream of a C-terminal HRV3C protease cleavage site, a monomeric Fc tag, and an 8XHisTag. Similarly, to express the SARS-CoV RBD-SD1, residues 306 to 577 of SARS-CoV S (Tor2 strain) were cloned upstream of a C-terminal HRV3C protease cleavage site, a monomeric Fc tag, and an 8XHisTag. Dimers are prepared on the basis of the molar ratio (2:1) of the analyte protein and fluorochrome-conjugated Strep-Tactin, respectively. PE (IBA Lifesciences, 6-5000-001) or allophycocyanin (APC) (IBA Lifesciences, 6–5010-001) conjugated Strep-Tactin was reacted with the protein over five additions, incubating for 15 min between each addition. The final concentration of tetramer was calculated with respect to the analyte protein. The solution was aliquoted on the basis of the expected usage per experiment, snap-frozen, and stored at −80°C. For quality control, monoclonal Abs were bound to polystyrene beads (Spherotech) per the manufacturer’s instructions. B cell hooks were tested on beads coated with Ab026204 and CH65; monoclonal antibody (mAb) Ab026204 binds SARS-CoV-2, and mAb CH65 binds influenza hemagglutinin (80) and was used a negative control. Briefly, 0.5 μl of beads was diluted to 25 μl in PBS and 0.02% NaN_3_. The bead mixture was added to 6 wells (25 μl per well) in a 96-well filter plate. B cell hooks were diluted to a final concentration of 40 μg/ml in 175 μl of PBS and 0.02% NaN_3_, and then, four serial twofold dilutions were made down to a final concentration of 5 μg/ml. Test conjugate dilutions (25 μl per well) were added to each bead set, with one additional well of each bead set left as an unstained control. After 30-min incubation, the solution was vacuumed off and the beads washed three times with 150 μl of PBS and 0.02% NaN_3_. The washed beads were resuspended in 125 μl of PBS and 0.02% NaN_3_, and the samples were run on a BD LSR II cytometer within 4 hours of preparation. Data were analyzed using FlowJo software (TreeStar), and plots of each dilution were compared with the unstained control.

### Antigen-specific B cell quantitation

S-specific B cells were assessed by flow cytometry as described (table S1) (39). Freshly isolated or archived (week 6) PBMCs or lymph node cells (2 × 10^6^ cells) were washed with PBS (Gibco) and pelleted by centrifugation at 500*g* for 7 min. Cells were resuspended in 150 μl of 1% BSA (Sigma-Aldrich) and 5 μM Chk2 inhibitor II in PBS, incubated 15 min at 4°C in the dark, and subsequently washed with PBS and 1% BSA (7 min at 500*g*). The cell pellet was stained with Abs described in table S2 for 30 min in the dark at 4°C. Total CD20^+^ and/or memory CD27^+^ S-specific B cells were identified as double-positive for biotinylated S-2P protein labeled with avidin-APC or avidin-PE (fig. S14). GC B cells were analyzed by flow cytometry (table S2) as detailed elsewhere (81). After staining, cells were washed as before and fixed with 1% paraformaldehyde before immediate acquisition on an LSRFortessa (BD Biosciences) using BD FACSDiva v.8.0 and analyzed with FlowJo software v10.7.1 (fig. S15).

### B cell ELISpot

Polyvinylidene fluoride membranes (Millipore) were activated with 70% ethanol for 1 min, subsequently coated with 1 μg of SARS-CoV-2 S protein (2019-nCoV) per well and blocked for 2 hours with 2% milk and stored at 4°C. Fresh or thawed cell preparations were washed and stimulated (10^6^ cells/ml) with R848 (1 μg/ml; InvivoGen) and human IL-2 (10 ng/ml; Miltenyi Biotec) for 72 hours in complete medium (cRPMI): RPMI 1640 medium (Gibco) containing penicillin, streptomycin, l-glutamine (Sigma-Aldrich), and 10% heat-inactivated FBS (Gibco). Stimulated cells (8 × 10^4^ per well) were incubated in triplicate wells of SARS-CoV-2 S protein–coated microtiter plates overnight at 37°C, washed with PBS and 0.05% Tween 20, and incubated 1 hour with biotinylated affinity-purified goat anti-human IgG (1 μg/ml; SouthernBiotech) at RT. Plates were washed with PBS and 0.05% Tween 20, incubated with a 1:4000 dilution of avidin-peroxidase (SouthernBiotech) for 1 hour at RT, and developed using the BD 3-amino-9-ethylcarbazole (AEC) kit using 100 μl per well. Dried membranes were analyzed with an automated ELISpot Reader System (Autoimmun Diagnostika GmbH). Results are reported as the number of ASC per 10^6^ mononuclear cells.

### T cell responses

Cryopreserved cells were thawed, or fresh preparations were washed and cultured in cRPMI (10^6^/ml) with or without overlapping peptides (2 μg/ml) spanning the length of SARS-CoV-2 S protein (JPT Technologies) or dimethyl sulfoxide (DMSO) vehicle together with costimulatory Abs against CD28 and CD49d from BD Biosciences. Naïve donor cells were included with each assay and, in addition to peptide pool or vehicle, were stimulated with 0.5× cell stimulation cocktail (eBiosciences) as a positive control. Cells were surface-stained as outlined in table S2 and then permeabilized with BD CytoFix/CytoPerm per the manufacturer’s recommendations and stained with intracellular Abs as described in table S2. Data were collected using LSRFortessa and BD FACSDiva v8.0 and analyzed with FlowJo software v10.7.1 (TreeStar). Intracellular cytokine gates (fig. S16) were Boolean-gated and reported as single positive events unless otherwise noted.

In addition, we performed the activation-induced marker (AIM) assay (49, 82) on cryopreserved LNC. Cells were thawed, rested 3 hours at 37°C with 5% CO_2_, resuspended in AIM V medium (Gibco), and transferred at 10^6^ cells per well to a 24-well plate. Cells were cultured with vehicle DMSO (negative control) or with SEB (0.5 μg/ml; Toxin Technologies) for 20 hours at 37°C with 5% CO_2_. After stimulation, cells were stained with described Abs per table S2 and acquired immediately on an LSRFortessa instrument running FACSDiva v8.0 software (BD Biosciences) and analyzed as described above (see fig. S17).

### Plasma cytokine measurements

IL-2, IL-4, IL-13, and IFN-γ in undiluted plasma samples were quantified by a custom 4-plex RM Luminex assay (Thermo Fisher Scientific) using protocols established by the supplier. ELISA results are reported as the average concentration of duplicate wells extrapolated from a standard curve.

### Statistical analyses

Spearman’s rank correlations were estimated between prespecified parameters at specific time points. Statistical analyses were performed using SAS version 9.4 (Cary, NC, USA). ID_50_ neutralization titer decline half-life was estimated on the basis of random-effects regression models of decay with first-order kinetics (83, 84). Models were fit separately by vaccine group, and data before the second vaccine dose (i.e., from weeks 0 and 4) were excluded. Biphasic decline was modeled using a linear spline with one knot (85), with different knots considered ranging from 8 to 18 weeks. For each vaccine group, the model with the knot at 18 weeks fit the best according to the Akaike Information Criterion.

## Data and materials availability:

All data needed to evaluate the conclusions in the paper are present in the paper or the Supplementary Materials. This work is licensed under a Creative Commons Attribution 4.0 International (CC BY 4.0) license, which permits unrestricted use, distribution, and reproduction in any medium, provided the original work is properly cited. To view a copy of this license, visit http://creativecommons.org/licenses/by/4.0/. This license does not apply to figures/photos/artwork or other content included in the article that is credited to a third party; obtain authorization from the rights holder before using such material.

## Supplementary Material

Suppl Table S3

Suppl Fig S1-S17 and Suppl Table S1-S2

## Figures and Tables

**Table 1.** Overview of study animals.

Vaccine group	Animal ID	Sex	Age at W0 (months)	Graphical symbol
**Protein+3M-052-SE**				Blue:
	RM 1	Male	2.6	Filled circle
	RM 2	Female	2.4	Open circle
	RM 3	Male	2.3	Filled triangle
	RM 4	Female	2.3	Open triangle
	RM 5	Female	2.2	Open inverted triangle
	RM 6	Male	2.1	Filled inverted triangle
	RM 7	Female	2.0	Open diamond
	RM 8	Male	1.6	Filled diamond
**mRNA-LNP**				Red:
	RM 9	Female	2.5	Open circle
	RM 10	Male	2.4	Filled circle
	RM 11	Male	2.4	Filled triangle
	RM 12	Female	2.3	Open triangle
	RM 13	Male	2.2	Filled inverted triangle
	RM 14	Female	2.1	Open inverted triangle
	RM 15	Male	2.1	Filled diamond
	RM 16	Female	2.1	Open diamond

**Table 2.** mRNA-1273 vaccine–induced immune responses in adult NHP and humans.

Study*	Immunogenicity			
	S-specific binding Abs^†^	Neutralizing Abs		T cell responses
		Pseudovirus^‡^	Live virus	
**Current infant NHP study (30 μg)**				
4 weeks after second dose	AUC (log_10_): 7.0 GMT: 55,564	ID_50_: 3174	ID_50_: 12,068	T_H_1; IL-21 ^+^ T_H_
10 weeks after second dose	AUC (log_10_): 6.2 GMT: 19,054	ID_50_: 1199	ID_50_: 1379	
**Adult NHP**				
Corbett *et al., N. Engl. J. Med*. 2020; 383(16):1544–1555				
4 weeks after second dose				
10 μg	AUC: 8421^§^	ID_50_: 103^§^	ID_50_: 501^§║^	T_H_1 > T_H_2; IL-21 ^+^ T_FH_
100 μg	AUC: 36,186^§^	ID_50_: 1862^§^	ID_50_: 3481^§║^	
Corbett *et al*., bioRxiv; doi.org/10.1101/2021.04.20.440647				
4 weeks after second dose				
30 μg	GMT: 64,000^¶^	ID_50_: ~10^3¶^		T_H_1 > T_H_2; IL-21 ^+^ T_FH_
**Human adults (18–55 years)**				
Jackson *et al., N. Engl. J. Med*. 2020; 383(20):1920–1931				
4 weeks after second dose				
10 μg	GMT: 299,751^§^	ID_50_: 81^§^		
100 μg	GMT: 782,719^§^	ID_50_: 232^§^		
250 μg	GMT: 1,192,154^§^	ID_50_: 270^§^		
2 weeks after second dose				
10 μg	GMT: 379,764^§^	ID_50_: 112^§^	ID_80_: 340^§^#	
100 μg	GMT: 811,119^§^	ID_50_: 344^§^	ID_80_: 654^§^#	
250 μg	GMT: 994,629^§^	ID_50_: 332^§^	No data	
Anderson *et al., N. Engl. J. Med*. 2020; 383(25): 2427–2438				
4 weeks after second dose				
25 μg	GMT: 323,945^§^			
100 μg	GMT: 1,183,066^§^	ID_50_: 402^§^	ID_50_: 878^§║^	T_H_1>T_H_2
Widge *et al., N. Engl. J. Med*. 2021; 384(1):80–82				
3 months after second dose				
100 μg	GMT: 235,228^¶^	ID_50_: 182^#^	ID_50_: 775^║#^	

* Study listed with reference, time of immune analysis for reported data, and vaccine dose.

† Plasma binding IgG levels against S protein are reported as AUC or geometric mean titers (GMT). Note that the SARS-CoV-2 strain from which the S antigen for the assay was derived is listed under each study.

‡ Lentiviral-based pseudovirus neutralization (PsVNA).

§ Wuhan.Hu.1 S protein.

║ Focus reduction neutralization test.

¶ D614G S protein.

# SARS-CoV-2 plaque-reduction neutralization testing.
