# Supplementary material for: SARS-CoV-2 vaccines elicit durable immune responses in infant rhesus macaques
Source: Sci Immunol. Author manuscript; Available in PMC 2022 Jan 20. (PMC8774290; doi:10.1126/sciimmunol.abj3684)
Supplement: Suppl Fig S1-S17 and Suppl Table S1-S2 [file NIHMS1770510-supplement-Suppl_Fig_S1-S17_and_Suppl_Table_S1-S2.pdf]

Science Immunology
AAAS

Supplementary Materials for

SARS-CoV-2 vaccines elicit durable immune responses in infant rhesus macaques

Carolina Garrido *et al.*

Corresponding author: Kristina De Paris, abelk@med.unc.edu

Published 15 June 2021, *Sci. Immunol.* **6**, eabj3684 (2021)
DOI: 10.1126/sciimmunol.abj3684

The PDF file includes:

Figs. S1 to S17
Tables S1 and S2

Other Supplementary Material for this manuscript includes the following:

Table S3

Supplementary Figures

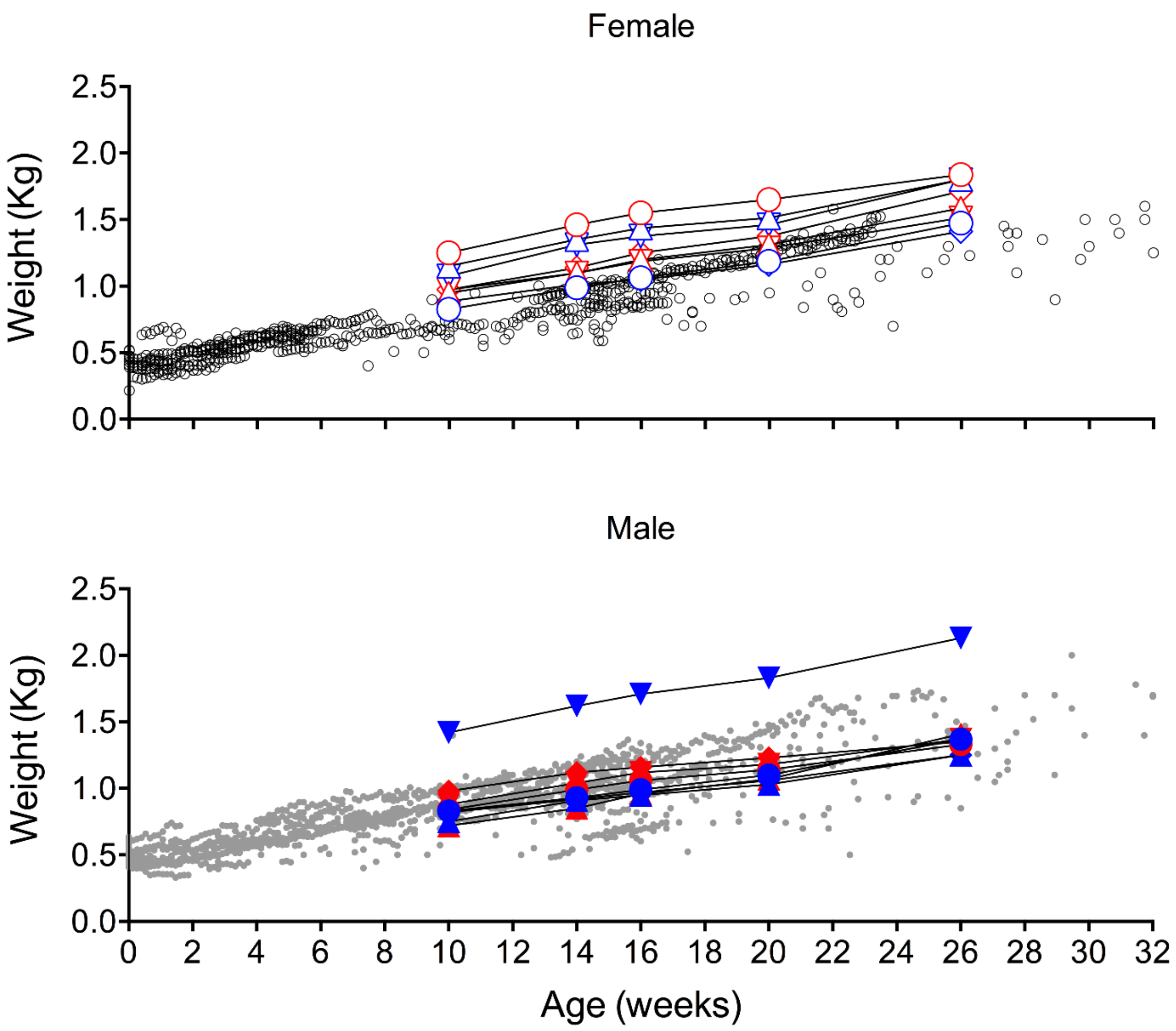

Fig. S1. Weight gain of SARS-CoV-2 immunized infant rhesus macaques.

Longitudinal weight data for all female (top) and male (bottom) animals from this study with their respective symbol shapes and colors (Table 1) overlaid with historical weight data from age and sex-matched infant rhesus monkeys housed outdoors at the CNPRC, Davis California (open black circles- female; filled gray circles- male).

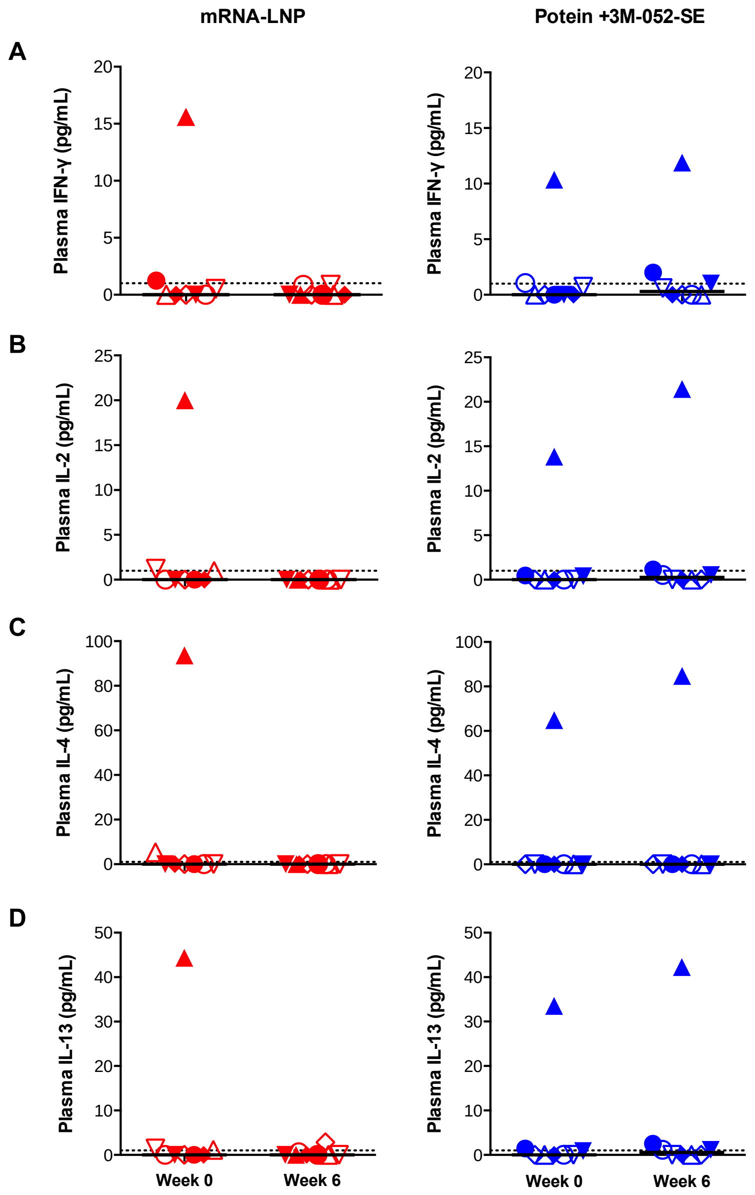

Figure S2. Th1 and Th2 cytokines in plasma of infant rhesus macaques prior to and following SARS-CoV-2 vaccination.

Plasma levels of IFN-γ (**panel A**) IL-2 (**panel B**), IL-4 (**panel C**) and IL-13 (**panel D**) were measured by multiplex Luminex assay. Different symbols represent individual animals in the mRNA-LNP (red) or Protein+3M-052-SE (blue) vaccine groups, respectively (Table S1). Horizontal lines represent median values.

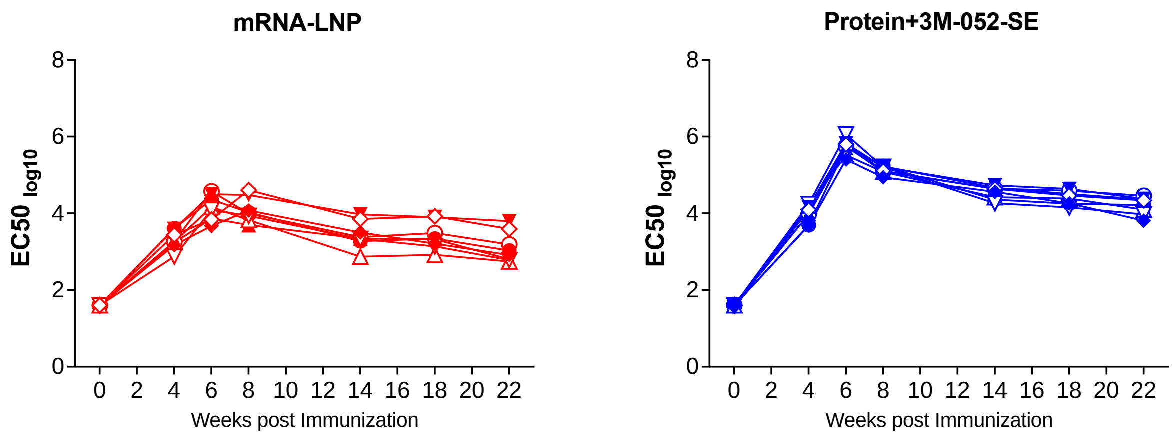

Figure S3. Plasma spike-specific IgG.

Plasma IgG binding responses were assessed by ELISA using serial dilutions of plasma Data are reported as EC50 values. At week 0, EC50 values corresponding to the lowest dilution were applied instead of a “0” value for non-binding antibodies. Different symbols represent individual animals in the mRNA-LNP (red) or Protein+3M-052-SE (blue) vaccine groups, respectively (Table 1).

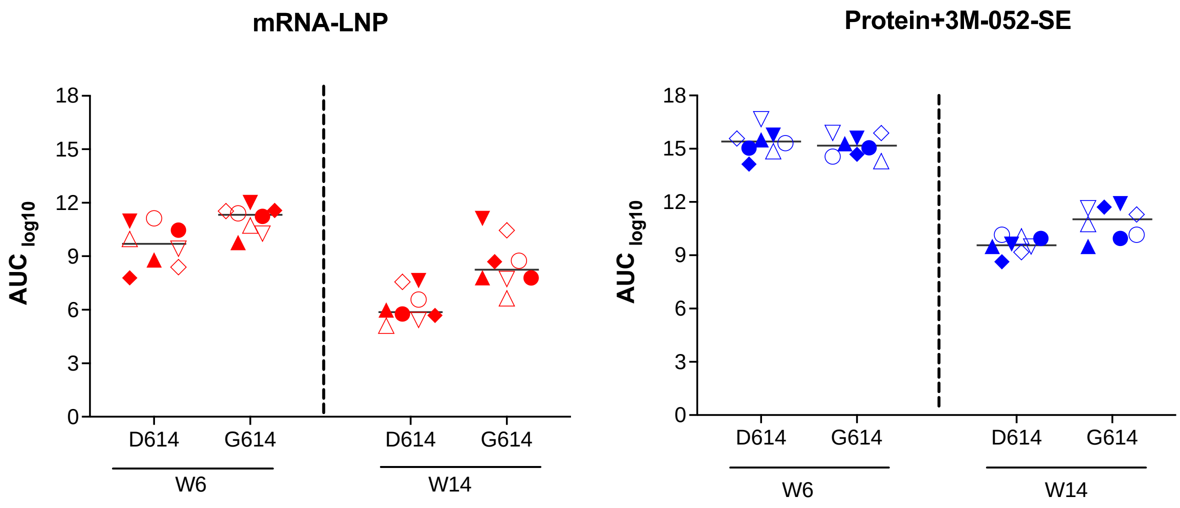

Figure S4. IgG binding to the D614 spike protein and the D614G variant.

Plasma IgG binding response was assessed by ELISA using serial dilutions of plasma collected at W6 and W14. Area under the curve (AUC) was compared between binding to D614 spike protein, the immunogen in both vaccines, and the G614 spike protein that was used in the neutralization assays because it represented the dominant variant at study initiation. Different symbols represent individual animals in the mRNA-LNP (red) or Protein+3M-052-SE (blue) vaccine groups, respectively (Table 1). Horizontal lines represent median values.

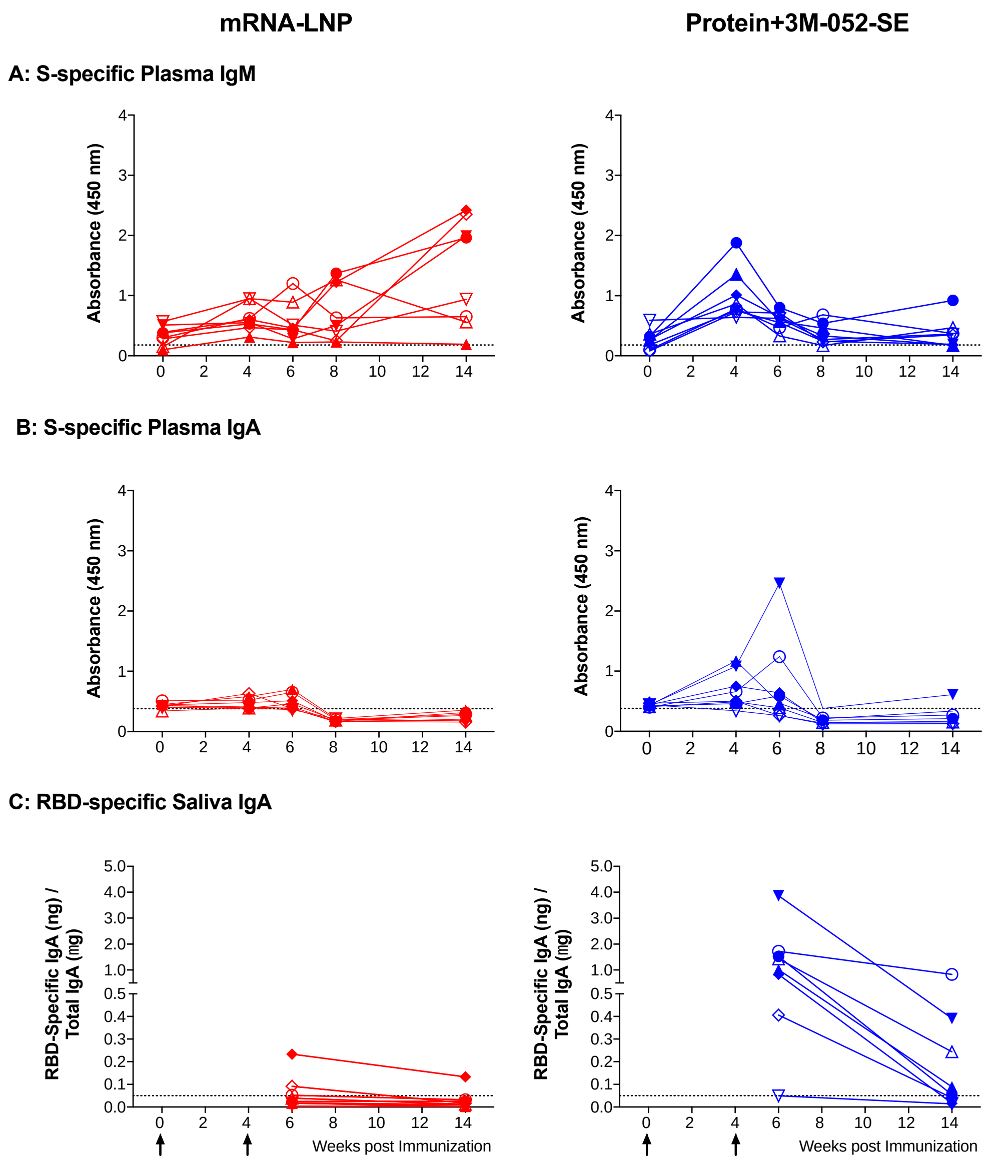

Figure S5. Plasma IgM and IgA and salivary IgA responses in infant rhesus macaques.

Panels A and B illustrate longitudinal plasma S-specific IgM (Panel A) and IgA (Panel B) levels measured by ELISA in 1:10 diluted plasma samples, reported as absorbance readings at 450 nm. In **Panel C**, we report receptor-binding domain-specific salivary IgA concentrations in ng per µg of total IgA. Note that salivary IgA was only measured at weeks 6 and 14. Red or blue lines and symbols represent the mRNA or protein vaccine groups, respectively, with different symbols representing individual animals (Table 1). In Panel C, the times of immunizations are indicated by arrows. The x-axis in all graphs lists the weeks after the first vaccine dose.

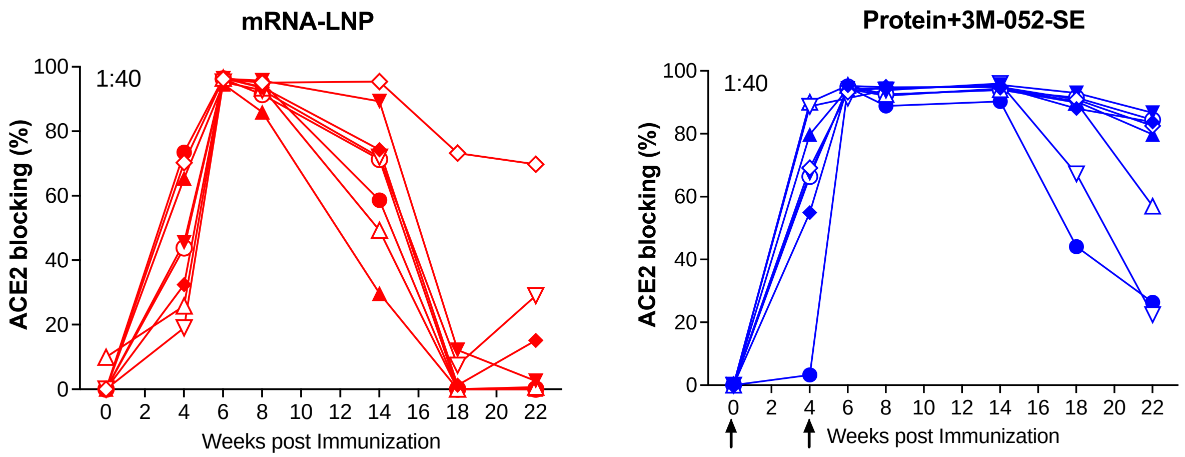

Figure S6. Plasma ACE2 blocking responses.

Capacity of plasma antibodies to mediate blocking of the RBD-ACE2 interaction was measured with an ELISA-based ACE2 blocking assay performed at 1:40 of vaccinated animals. Graph shows results as %ACE2 blocking in the mRNA-LNP (red) or Protein+3M-052-SE vaccine (blue) groups over time, with different symbols representing individual animals (Table 1).

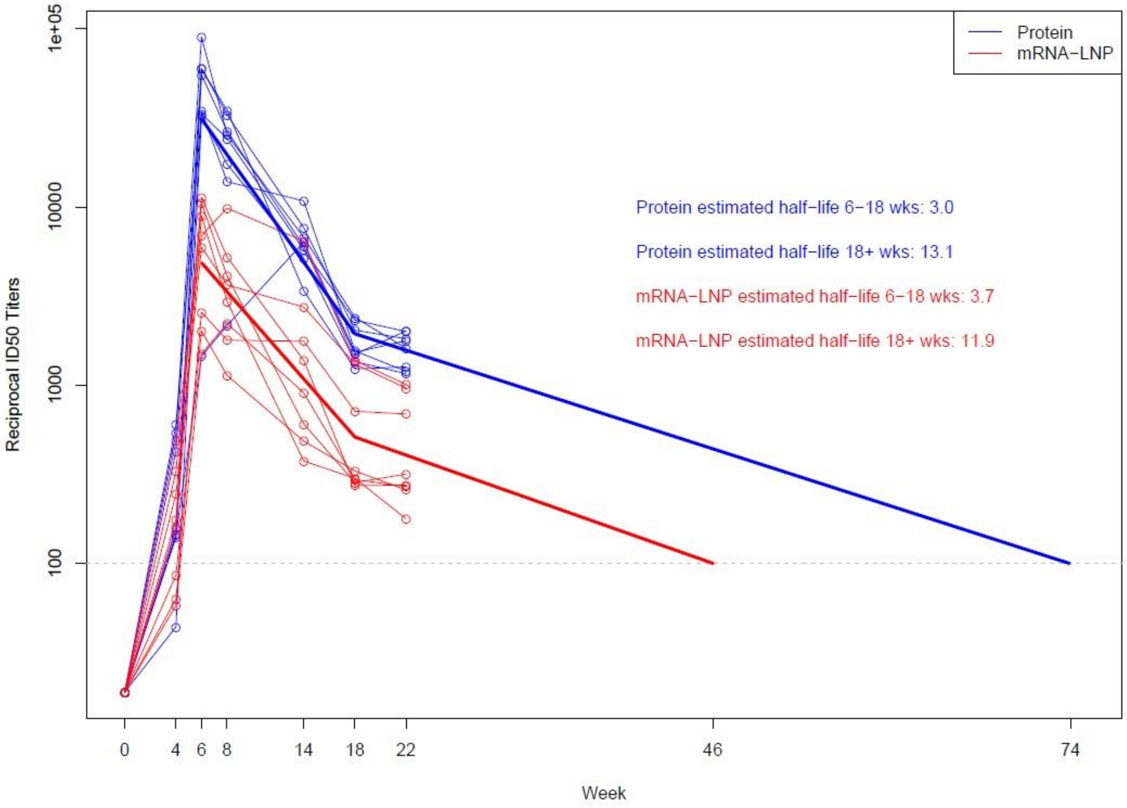

Figure S7. Modelled bi-phasic decline of neutralizing ID_{50} titers.

ID_{50} neutralization titer (obtained using the pseudovirus neutralization assay) decline half-life was estimated based on random-effects regression models of decay with first-order kinetics. Models were fit separately by vaccine group and data prior to the second vaccine dose (i.e., from weeks 0 and 4) were excluded. Bi-phasic decline was modeled using a linear spline with one knot, with different knots considered ranging from 8 to 18 weeks. For each vaccine group, the model with the knot at 18 weeks fit the best according to the Akaike Information Criterion.

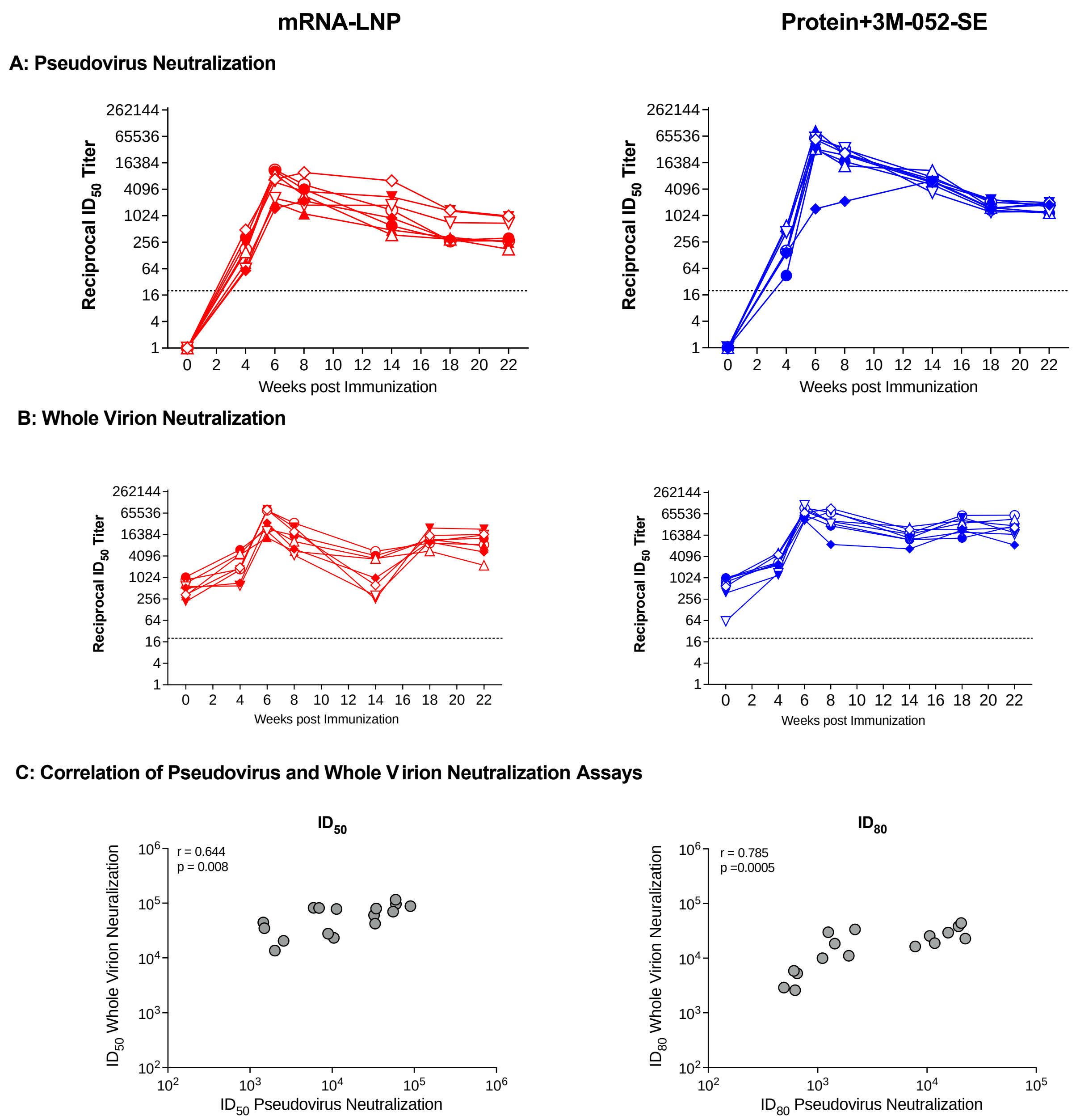

Figure S8: Vaccine-elicited SARS-CoV-2 neutralization responses.

Panel A shows the ID_{50} neutralization titers obtained using Spike-psudotyped viruses in 293T/ACE2 cells, and expressing the results as reciprocal 50% inhibitory dilution (ID_{50}). **Panel B** shows the ID_{50} neutralization titers obtained using a whole virus neutralization assay performed with Vero E6 cells. Dashed lines in Panels A and B indicate the detection cutoff. **Panel C** illustrates the correlation of ID_{50} (left graph) and ID_{80} (right graph) neutralizing titers from both vaccine groups obtained in the pseudovirus or whole virus neutralization assay at week 6. Correlation was assessed using Spearman rank test.

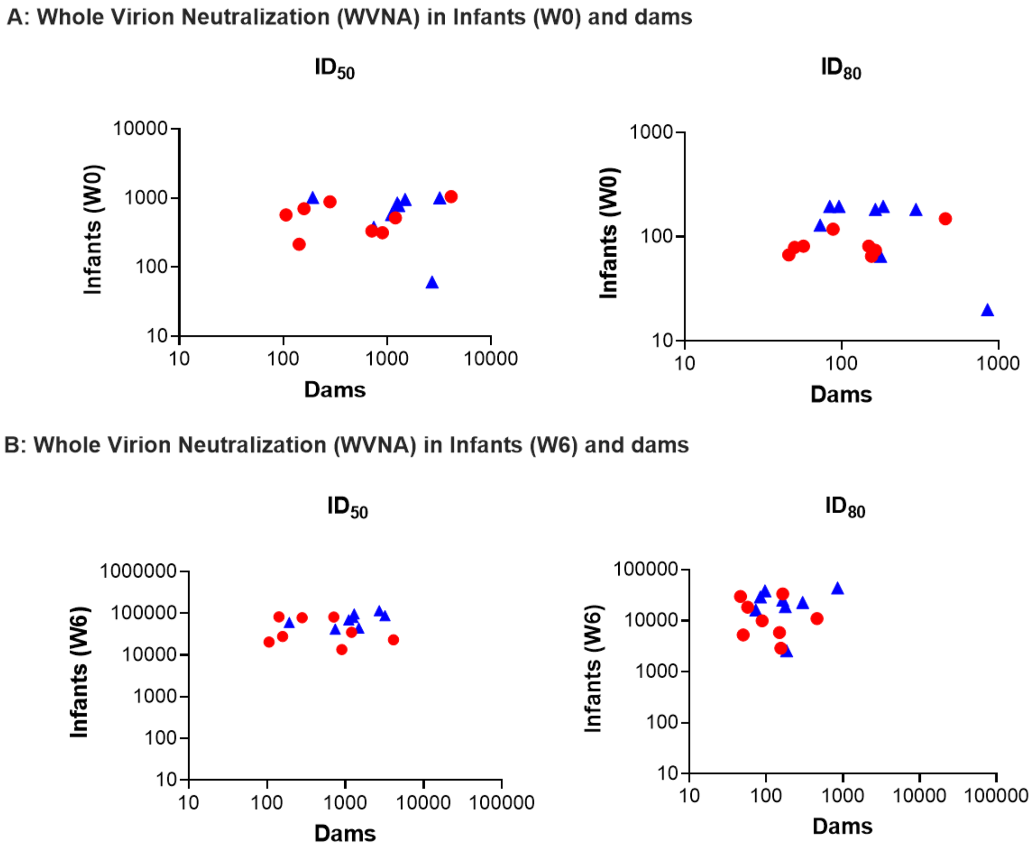

Figure S9. Correlation of whole virion neutralization in dams and infants.

Whole virion neutralization (WVNA) was measured in dams and in infants before the vaccine (W0) and after the vaccine boost (W6). There was no correlation of ID_{50} or ID_{80} neutralization titers between infants and dams (Spearman rank analysis). Blue symbols correspond to protein vaccine and red symbols to mRNA-LNP.

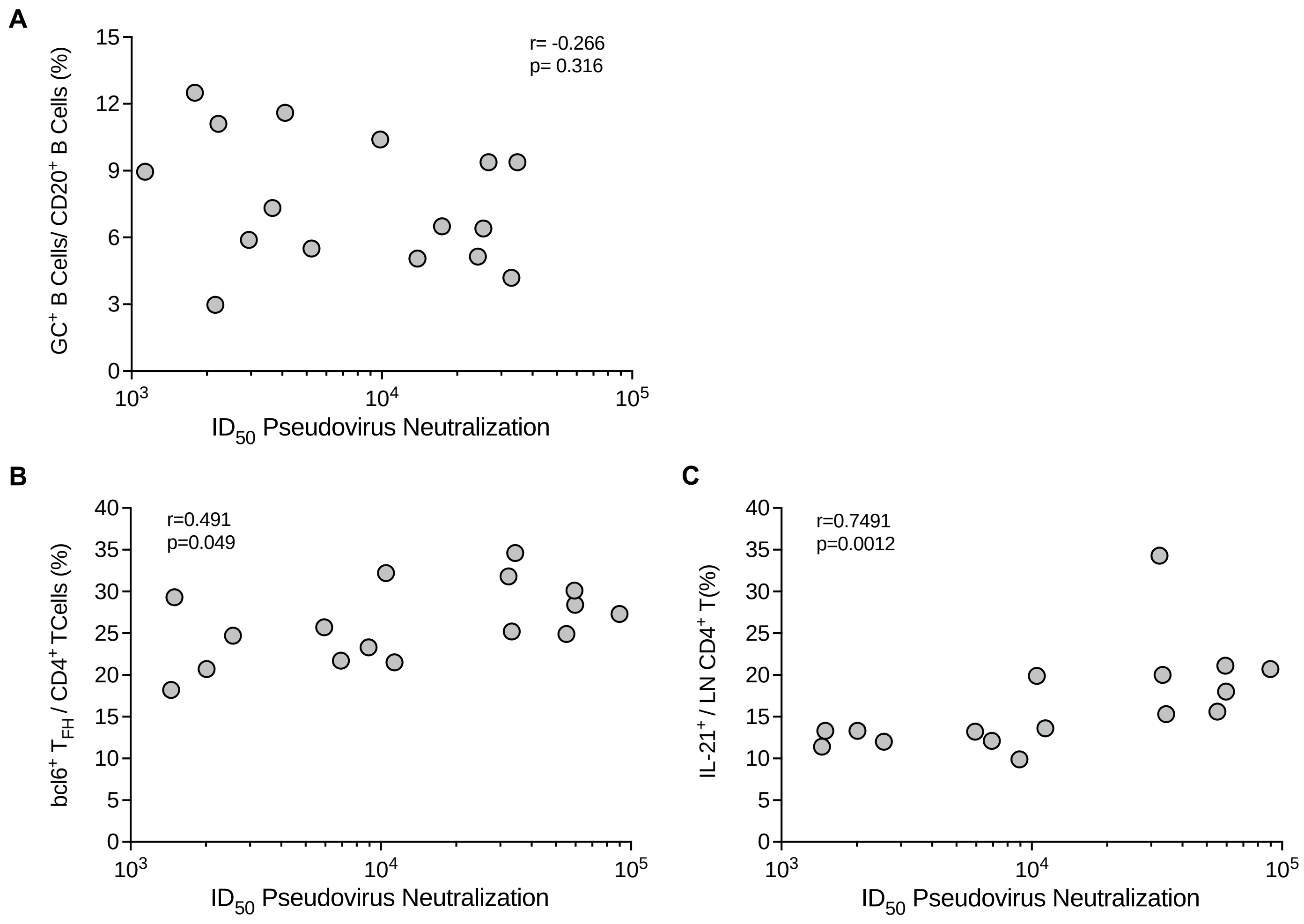

Figure S10. Correlation between neutralizing antibody titers at week 6, germinal center B cells, follicular T helper cells, and IL-21$^+$ lymph node CD4$^+$ T cells.

Pseudovirus neutralizing antibody ID_{50} titers at week 6 did not correlate with the frequency of GC B cells **(A)** or bcl6+ T_{FH} **(B),** but positively correlated with lymph node CD4$^+$IL-21$^+$ T cells **(C)**. Each symbol represents a single animal, animals from both vaccine groups were included in the analysis. Correlations were assessed by Spearman rank test.

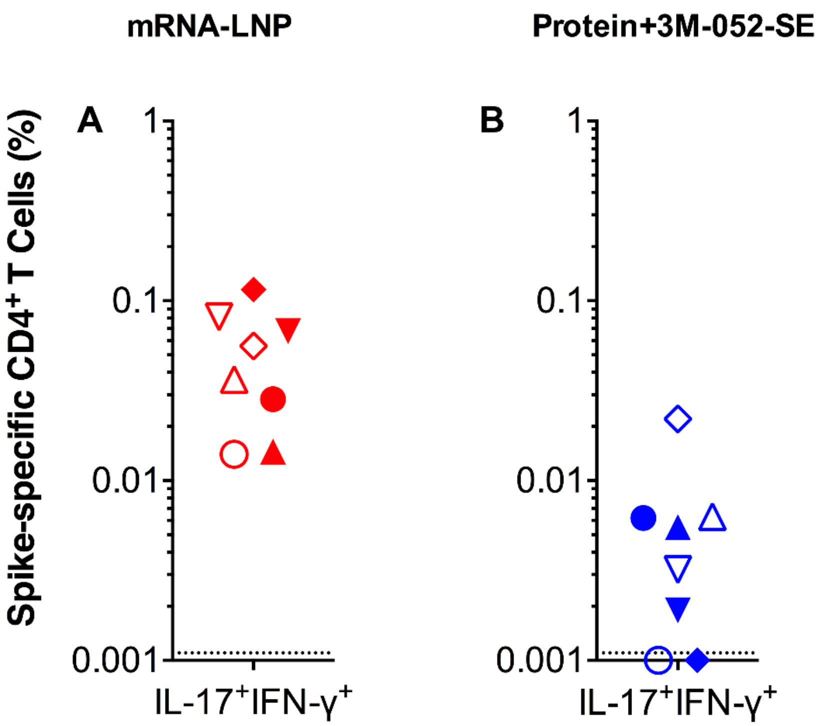

Figure S11: Polyfunctional CD4$^+$ T cells in SARS-CoV-2 immunized infant rhesus macaques at week 14.

Panels A and B show the frequencies of CD4$^+$ T cells that co-produced IL-17 and IFN-γ in response to stimulation with spike protein overlapping peptides in animals of the mRNA-LNP or Protein-3M-052-SE vaccine group, respectively. Individual symbols represent individual animals in each group (Table 1).

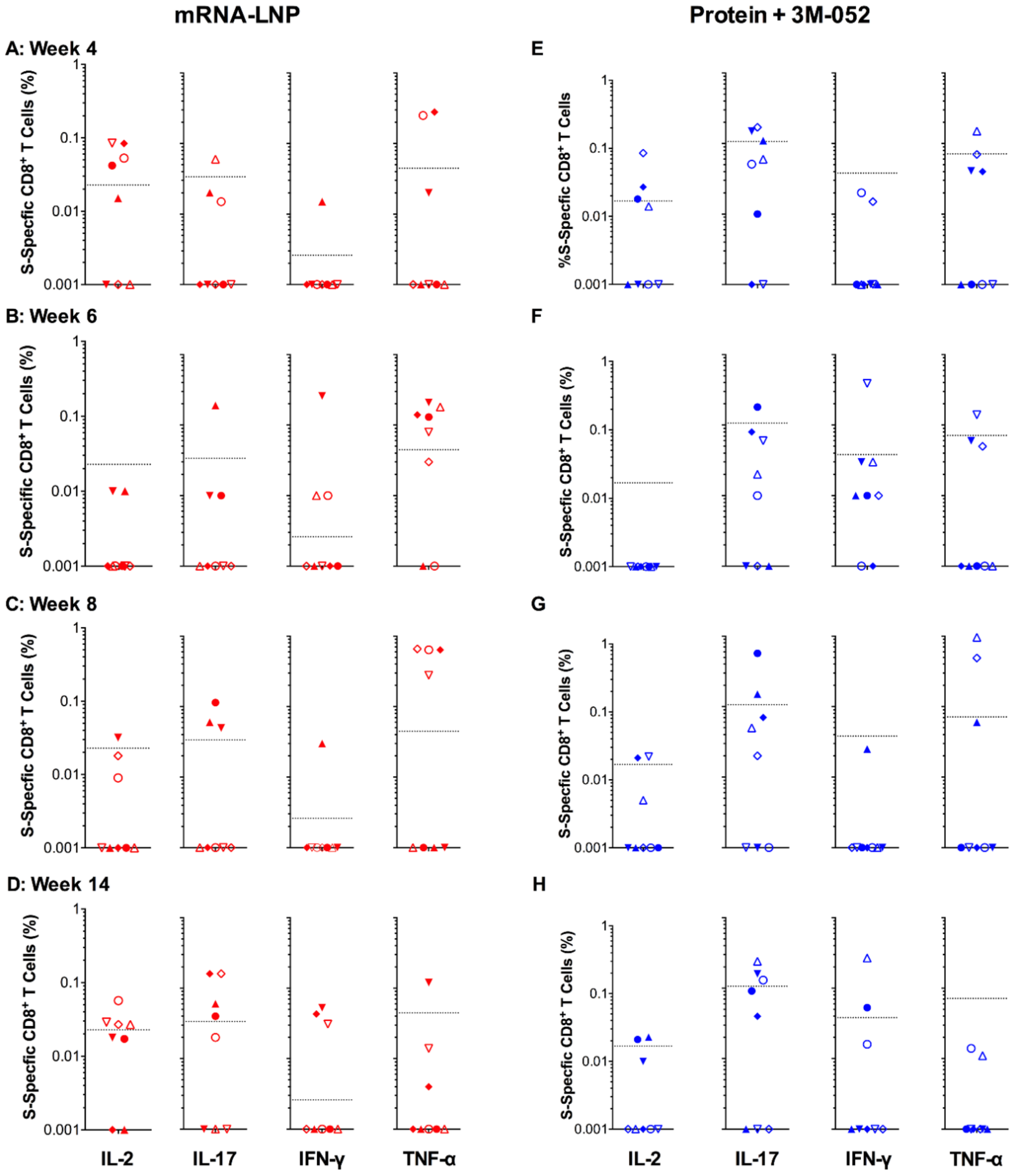

Figure S12. Spike-specific CD8^{+} T cell responses in SARS-CoV-2 immunized infant rhesus macaques. Intracellular cytokine staining was performed as described in Figure 5 at weeks 0, 6, 4, 8, and 14 to assess CD8^{+} T-cell responses. **Panels A, B, C and D** show responses detected in PBMC from the mRNA-LNP group at weeks 4, 6, 8, and 14, respectively. **Panels E, F, G and H** show responses in Protein+3M-052+SE vaccinees at weeks 4, 6, 8 and 14, respectively. The legend and symbols used mirror those from Figure S11

(see also Table 1). Dotted lines represent the cut-off for cytokine-positive responses, determined as 2 standard deviations above median values of SARS-CoV-2 naïve animals.

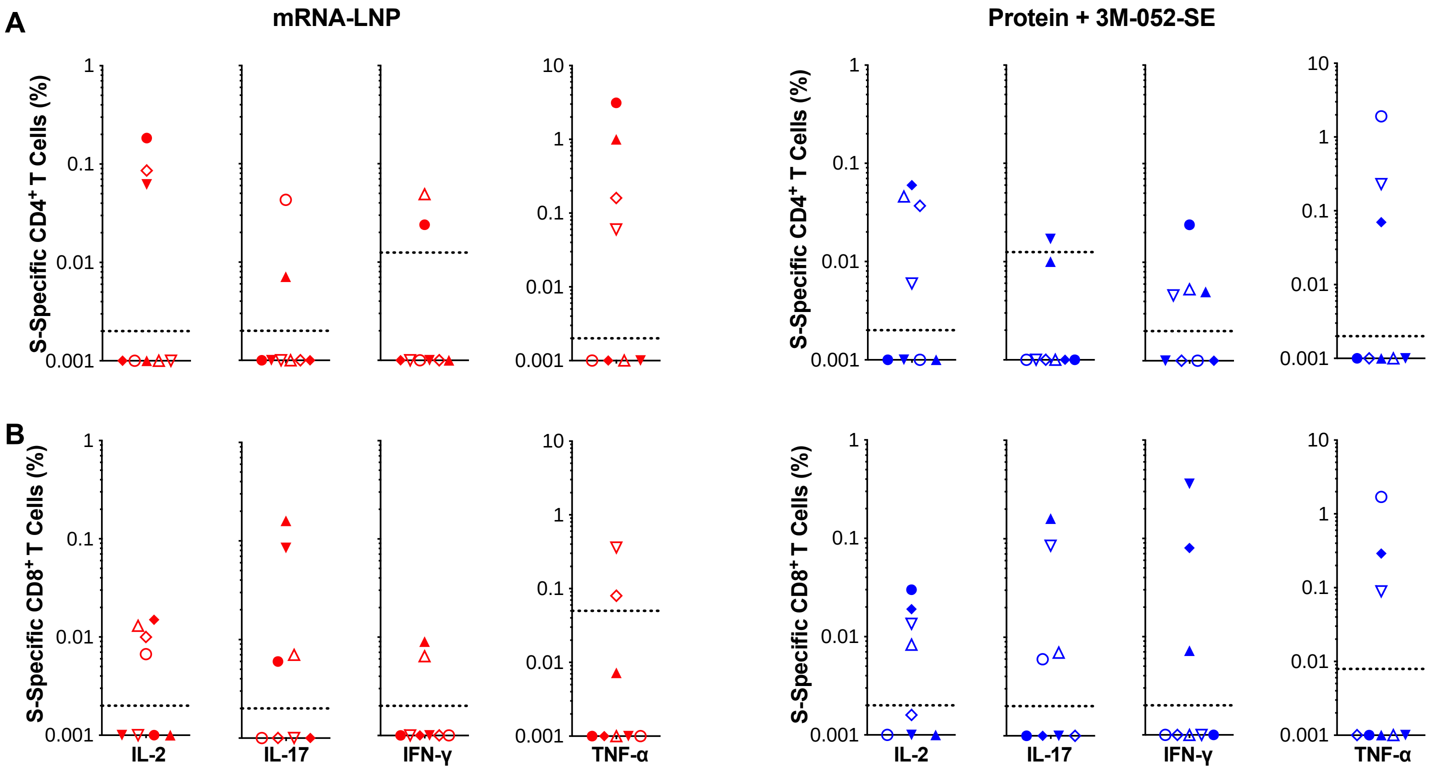

Figure S13. Spike-specific T cell responses in LN following SARS-CoV-2 vaccination.

Intracellular cytokine staining in lymph node biopsy samples at week 6 to measure Spike-specific T cell responses. **Panel A** shows mRNA-LNP and Protein+3M-052+SE CD4^{+} T-cell responses, respectively. **Panel B** represents CD8^{+} T cell cytokine responses from mRNA-LNP and Protein+3M-052+SE animals, respectively. Symbols and the legend mirror those from Figure S12 (see also Table 1). Dotted lines represent 2 standard deviations from SARS-CoV-2 naïve LN samples.

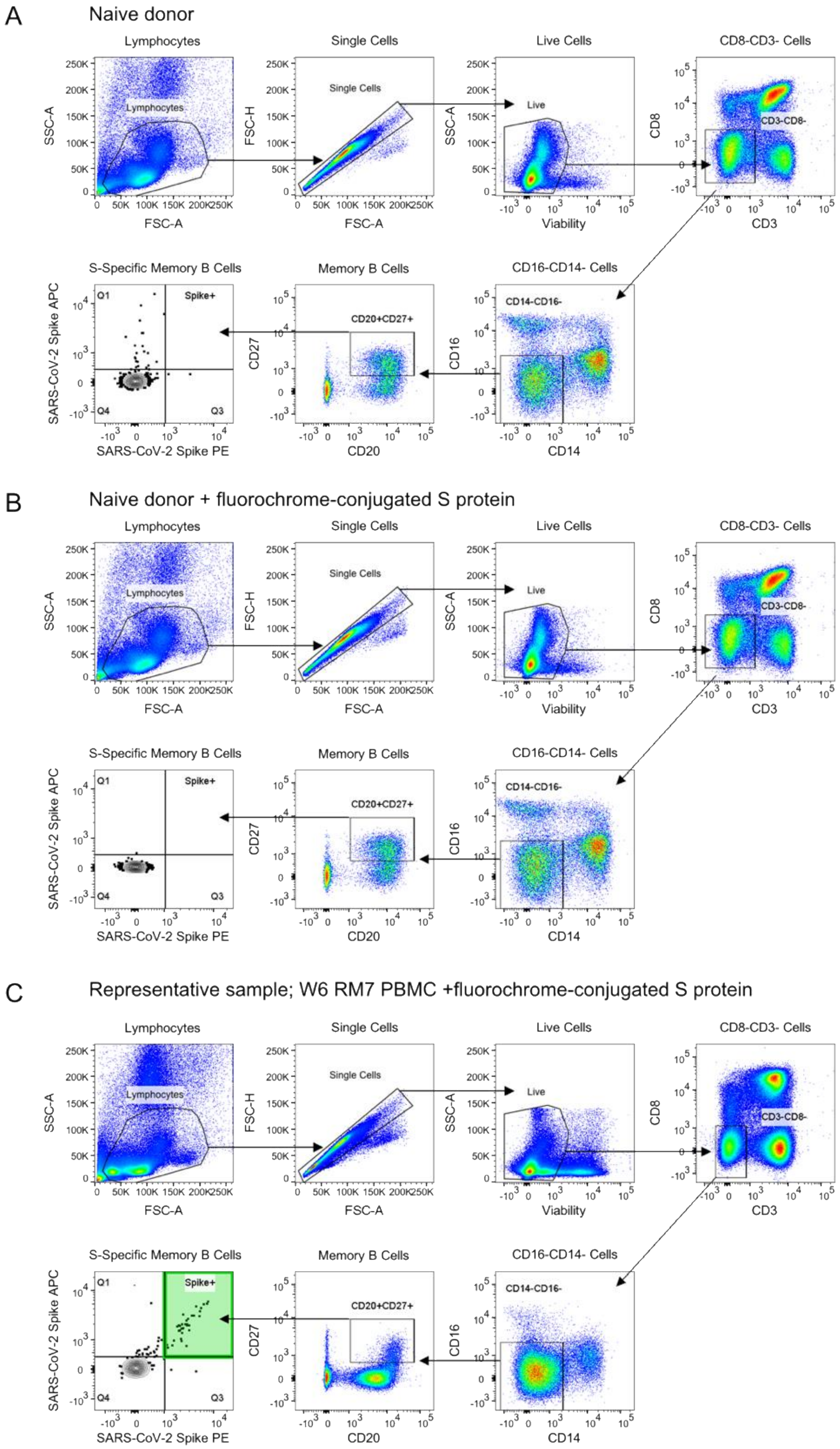

Figure S14. S-specific B cell gating strategy.

Panel A shows a representative plot from RM7; **Panel B** shows a naive, age-matched control macaque without SARS-CoV-2 S protein conjugates; **Panel C** shows the same donor macaque with the SARS-CoV-2 S protein conjugates included.

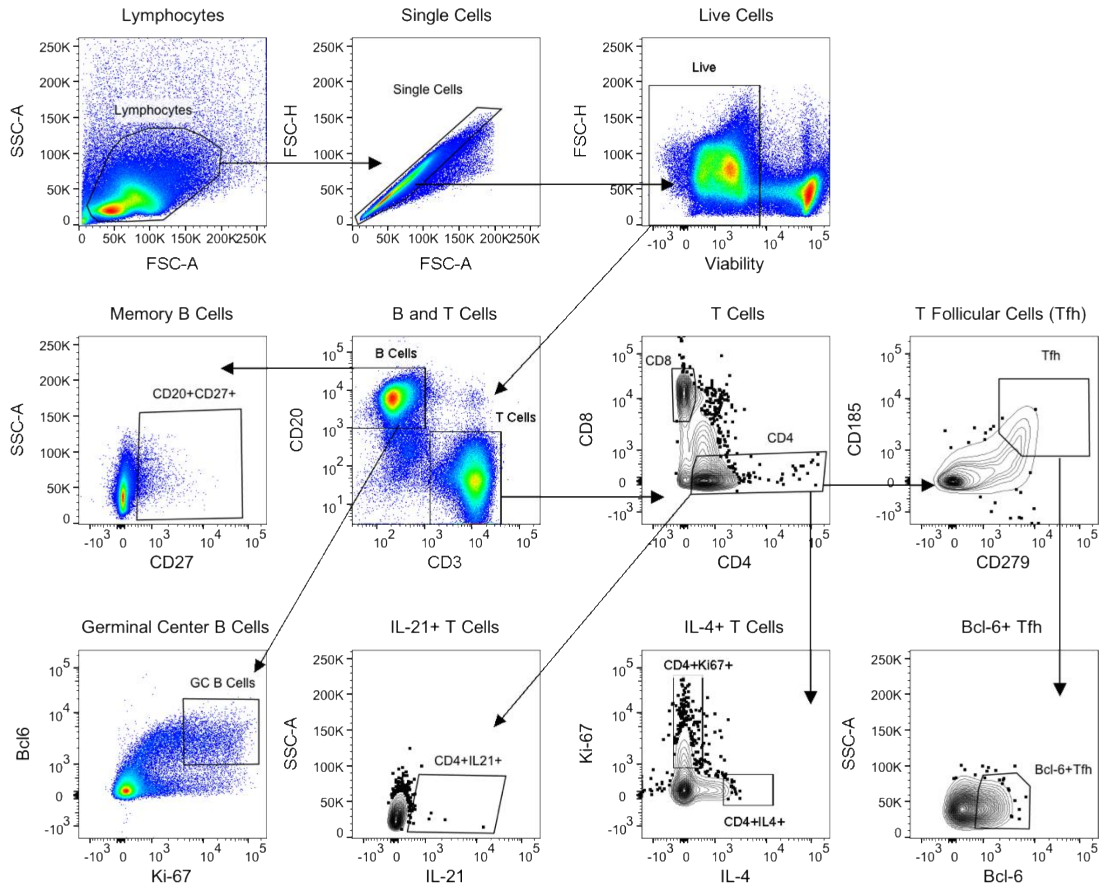

Figure S15. Gating strategy for lymph node germinal center B cells and T cell populations.

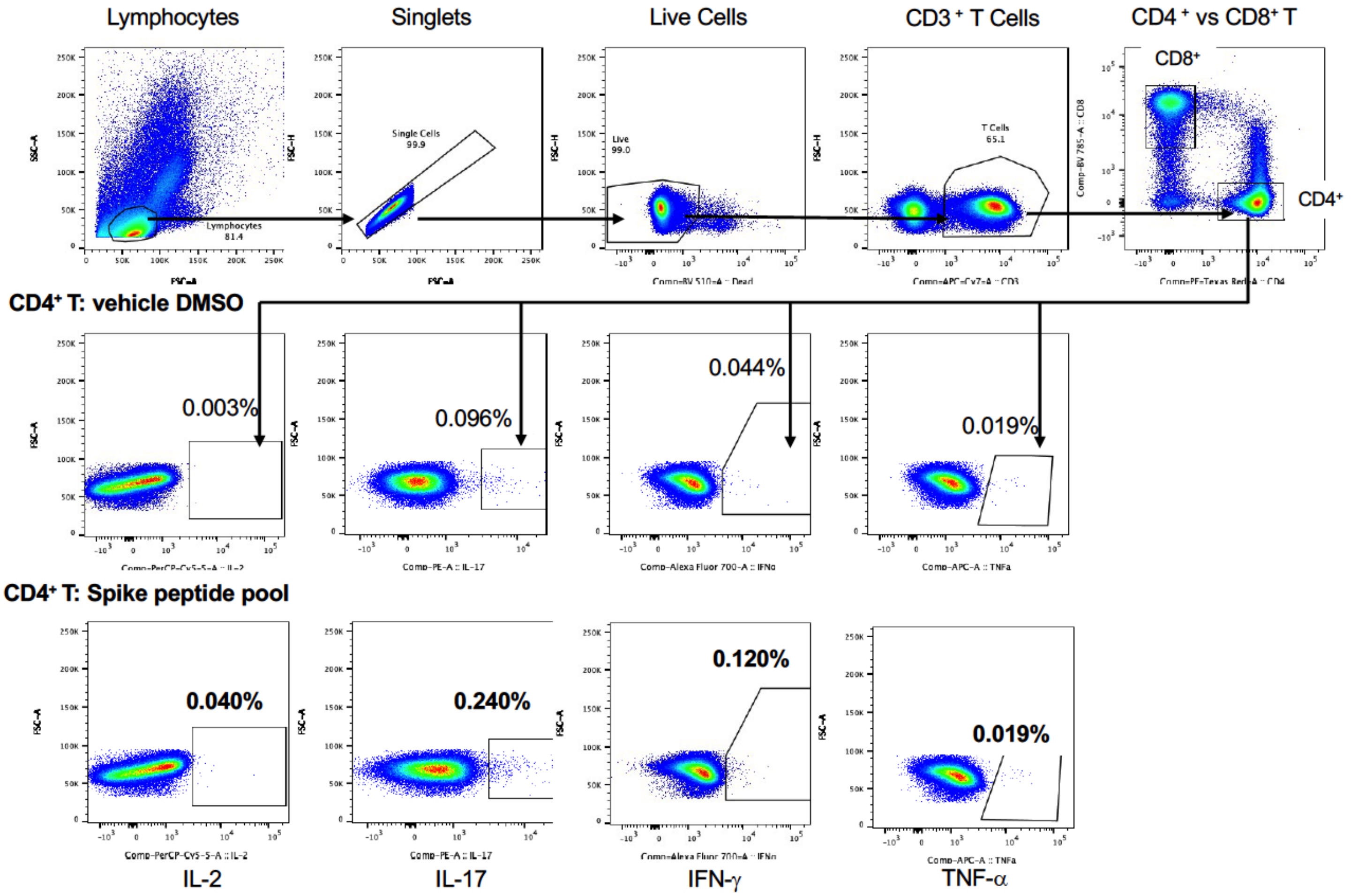

Figure S16: Gating strategy for intracellular cytokine staining of spike-specific T cells. Representative example of a week 14 sample. Lymphocytes were gated for singlets, live cells, and then for $CD3^+$ T cells and subsequently for $CD4^+$ and $CD8^+$ T cell populations. $CD4^+$ T cell population positive for IL-2, IL-17, IFN-γ, or TNF-α production after stimulation with vehicle control (DMSO; middle panel) or the Spike protein peptide pool (bottom panel) are shown. The percent of cytokine positive T cells in indicated in each plot.

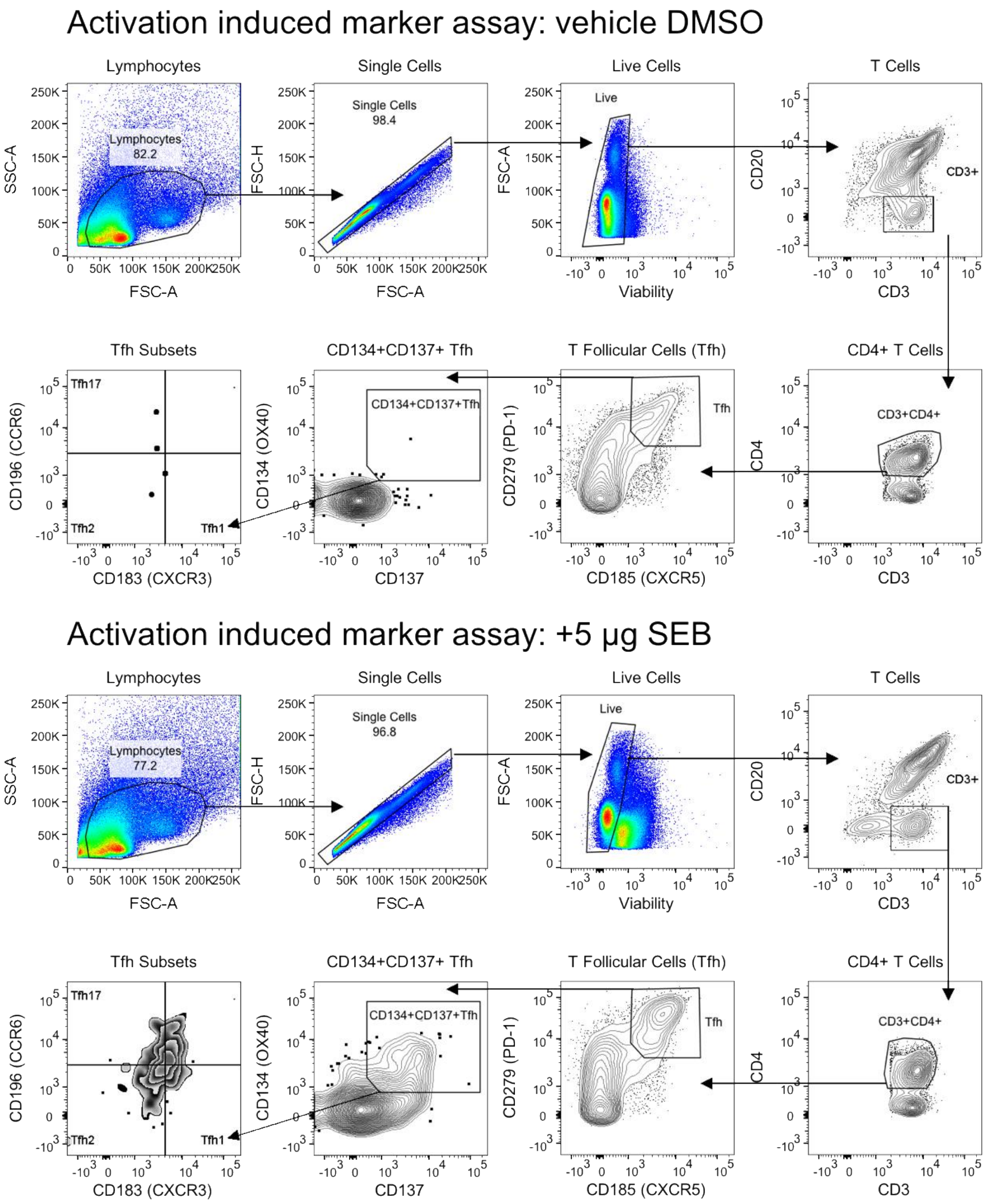

Figure S17. Activation-induced marker assay gating strategy for follicular T helper cells.

Top two rows: DMSO vehicle control cultures; bottom two rows: staphylococcal enterotoxin B-stimulated samples.

Supplementary Tables

Table S1: CBC reference values (A) for infant rhesus macaques age-matched to study animals immunized with (B) S-2P Protein+3M-052-SE or (C) S-2P mRNA-LNP vaccine.

A

Reference Values (range)																		
WBC $x10^3$/µl	RBC $x10^6$/µl	Hgb gm/dl	HCT %	MCV fl	MCH pg	MCHC pg/fl	Plts $x10^5$/µl	Segm %	Segm /µl	Lympho %	Lympho /µl	Mono %	Mono /µl	Eos %	Eos /µl	Baso %	Baso /µl	Plasma protein gm/dl
7.4-13.0	4.91-5.69	11.0-12.8	34.2-39.6	66-74	20.9-24.1	31.5-33.1	3.54-6.52	25-47	1892-5418	49-72	4172-8114	1.0-5.0	69-626	1-3	62-283	1.0-1.48	64-142	6.2-6.9

B

Protein+3M-052-SE																				
Animal	Study Week	WBC $x10^3$/µl	RBC $x10^6$/µl	Hgb gm/dl	HCT %	MCV fl	MCH pg	MCHC pg/fl	Plts $x10^5$/µl	Segm %	Segm /µl	Lympho %	Lympho /µl	Mono %	Mono /µl	Eos %	Eos /µl	Baso %	Baso /µl	Plasma protein gm/dl
	0	8.00	5.32	12.50	38.5	72	23.50	32.40	6.73	34	2720	63	5040	2	160	1	80	0	0	6.50
	4	10.80	5.31	12.00	37.60	71	22.60	32.00	4.68	59	6372	37	3996	4	432	0	0	0	0	6.80
RM 1	6	9.20	5.17	11.40	36	70	22.10	31.80	4.08	48	4416	46	4232	6	552	0	0	0	0	6.70
	8	5.30	5.18	11.50	36.1	70	22.20	31.90	4.79	24	1272	72	3816	3	159	0	0	1	53	6.70
	14	8.20	6.21	13.20	42.1	68	21.30	31.50	4.17	37	3034	60	4920	2	164	1	82	0	0	7.50
	0	6.40	5.31	12.30	38.0	72	23.10	32.20	3.30	23	1472	67	4288	4	256	6	384	0	0	6.50
	4	7.70	5.81	12.80	40.9	70	22.00	31.20	3.81	23	1771	76	5852	1	77	0	0	0	0	6.50
RM 2	6	11.10	5.37	12.10	37.7	70	22.50	32.10	0.96	25	2775	68	7548	4	444	3	333	0	0	6.60
	8	10.00	5.73	12.60	40.1	70	22.00	31.40	4.87	31	3100	64	6400	4	400	1	100	0	0	7.00
	14	9.00	6.03	12.90	41.3	68	21.40	31.30	3.99	23	2070	73	6570	1	90	3	270	0	0	6.70
	0	7.70	5.63	12.90	39.7	71	22.90	32.50	3.88	37	2849	55	4235	1	77	7	539	0	0	6.50
	4	8.30	5.73	12.70	39.6	69	22.10	32.00	2.65	25	2075	73	6059	0	0	2	166	0	0	6.50
RM 3	6	10.70	5.44	11.80	37.5	69	21.80	31.60	2.64	22	2354	73	7811	3	321	2	214	0	0	6.30
	8	9.50	5.60	12.20	38.3	68	21.70	31.80	2.72	38	3610	60	5700	2	190	0	0	0	0	6.40
	14	8.40	6.31	13.20	42.5	67	21.00	31.10	5.04	41	3444	57	4788	2	168	0	0	0	0	7.00
	0	8.00	5.16	12.20	38.3	74	23.70	31.90	5.59	17	1360	76	6080	1	80	6	480	0	0	6.00
	4	11.00	5.46	12.50	39.7	73	22.90	31.50	5.91	29	3190	68	7480	1	110	2	220	0	0	6.20
RM 4	6	9.50	4.31	9.50	30.5	71	22.00	31.10	2.64	8	760	85	8075	3	285	4	380	0	0	6.00
	8	15.70	5.64	12.60	40.0	71	22.20	31.40	3.36	14	2198	83	13031	1	157	2	314	0	0	6.60
	14	11.90	5.57	12.60	40.0	72	22.60	31.40	4.46	16	1904	83	9877	1	119	0	0	0	0	6.50
	0	11.70	6.09	13.50	41.4	68	22.20	32.60	4.67	31	3627	58	6786	6	702	5	585	0	0	6.60
	4	18.30	6.41	13.70	42.8	67	21.40	32.10	4.08	30	5490	60	10980	6	1098	4	732	0	0	7.00
RM 5	6	15.80	6.29	13.20	41.8	67	20.90	31.50	3.48	21	3318	64	10112	13	2054	2	316	0	0	6.80
	8	11.50	6.33	13.10	41.3	65	20.70	31.70	2.78	17	1955	74	8510	4	460	5	575	0	0	6.80
	14	16.30	6.50	13.30	42.5	65	20.50	31.30	4.42	22	3586	74	12062	3	489	1	163	0	0	7.10
	0	8.30	5.51	13.20	40.4	73	23.90	32.70	5.65	21	1743	72	5976	5	415	2	166	0	0	6.50
	4	11.60	6.00	13.90	42.8	71	23.10	32.40	4.59	13	1508	81	9396	1	116	5	580	0	0	6.90
RM 6	6	16.30	5.76	12.90	40.4	70	22.30	31.90	5.59	26	4238	70	11410	3	489	1	163	0	0	6.80
	8	8.20	5.96	13.20	41.1	69	22.20	32.10	2.87	22	1804	72	5904	4	328	2	164	0	0	6.50
	14	10.50	5.76	12.50	39.2	68	21.70	31.90	4.92	33	3465	62	6510	1	105	4	420	0	0	6.80
	0	4.30	4.57	10.90	33.4	73	23.80	32.50	4.65	19	817	77	3311	0	0	4	172	0	0	6.30
	4	5.80	4.88	11.40	35.6	73	23.30	32.00	3.27	24	1392	74	4292	1	58	1	58	0	0	6.10
RM 7	6	8.70	4.73	11.00	34.6	73	23.20	31.70	5.33	17	1479	77	6699	1	87	5	435	0	0	6.30
	8	5.60	4.90	11.30	35.6	73	23.10	31.90	3.67	30	1680	65	3640	0	0	5	280	0	0	6.10
	14	9.10	5.33	12.30	38.5	72	23.20	32.00	4.88	26	2366	68	6188	3	273	3	273	0	0	6.70
	0	8.80	4.92	12.30	37.3	76	25.00	33.00	4.94	12	1056	82	7216	4	352	2	176	0	0	6.50
	4	10.90	5.04	11.50	35.6	71	22.90	32.40	2.77	26	2834	74	8066	0	0	0	0	0	0	6.30
RM 8	6	9.80	4.93	11.10	34.7	70	22.50	32.00	4.75	9	882	86	8428	5	490	0	0	0	0	6.40
	8	10.90	5.40	12.40	38.5	71	22.90	32.20	4.05	18	1962	69	7521	10	1090	3	327	0	0	6.30
	14	9.20	5.61	13.00	40.3	72	23.10	32.20	2.95	18	1656	76	6992	3	276	3	276	0	0	6.60

C

mRNA-LNP																				
Animal	Study Week	WBC $x10^3$/µl	RBC $x10^6$/ul	Hgb gm/dl	HCT %	MCV fl	MCH pg	MCHC pg/fl	Plts $x10^5$/µl	Segm %	Segm /µl	Lympho %	Lympho /µl	Mono %	Mono /µl	Eos %	Eos /µl	Baso %	Baso /µl	Plasma protein gm/dl
	0	11.70	5.18	12.90	39.7	77	24.90	32.50	4.70	31	3627	65	7605	0	0	4	468	0	0	6.60
	4	11.20	5.08	12.40	37.4	74	24.40	33.10	4.12	23	2576	73	8176	1	112	3	336	0	0	6.50
RM 9	6	11.30	5.04	11.80	36.4	72	23.30	32.30	4.87	23	2599	71	8023	0	0	6	678	0	0	6.60
	8	10.60	5.34	11.90	37.5	70	22.20	31.60	4.77	26	2756	70	7420	3	318	1	106	0	0	7.40
	14	8.30	5.68	12.60	39.9	70	22.20	31.70	3.88	42	3486	54	4482	0	0	4	332	0	0	7.00
	0	8.00	5.57	12.70	39.3	71	22.90	32.40	4.07	29	2320	69	5520	1	80	1	80	0	0	6.60
	4	10.40	6.02	13.10	41.4	69	21.80	31.70	4.01	30	3120	64	6656	0	0	6	624	0	0	6.50
RM 10	6	10.50	5.84	12.80	39.8	68	21.80	32.00	3.83	17	1785	59	6195	8	840	16	1680	0	0	6.50
	8	8.40	5.80	12.60	39.5	68	21.80	32.00	4.35	21	1764	68	5712	5	420	6	504	0	0	7.10
	14	7.70	5.89	12.70	39.5	67	21.50	32.10	0.64	20	1540	75	5775	3	231	2	154	0	0	7.50
	0	8.40	5.52	12.90	39.9	72	23.30	32.30	2.35	30	2520	68	5712	0	0	2	168	0	0	6.60
	4	10.60	5.08	11.70	36.1	71	23.00	32.40	6.46	29	3074	67	7102	1	106	3	318	0	0	6.50
RM 11	6	10.30	4.63	10.60	33.1	72	22.90	32.10	8.44	20	2060	79	8137	1	103	0	0	0	0	6.20
	8	9.10	5.23	12.00	37.9	72	22.80	31.60	5.58	28	2548	64	5824	8	728	0	0	0	0	6.60
	14	7.60	5.32	11.90	37.7	71	22.40	31.60	4.28	26	1976	70	5320	3	228	1	76	0	0	6.60
	0	10.10	5.77	13.60	42.6	74	23.60	32.00	3.83	42	4242	55	5555	2	202	1	101	0	0	6.50
	4	10.30	5.58	12.90	40.3	72	23.20	32.10	4.92	43	4429	55	5665	0	0	2	206	0	0	6.50
RM 12	6	10.50	5.57	12.90	40.1	72	23.10	32.00	3.62	25	2625	69	7245	1	105	5	525	0	0	6.70
	8	8.20	5.52	12.70	40.4	73	23.00	31.40	3.94	28	2296	69	5658	2	164	1	82	0	0	6.40
	14	9.40	5.88	13.40	42.2	72	22.90	31.90	4.17	24	2256	72	6768	0	0	4	376	0	0	6.50
	0	13.00	5.77	12.60	40.0	69	21.90	31.60	5.79	24	3120	69	8970	3	390	4	520	0	0	6.60
	4	17.60	5.94	12.40	39.0	66	20.80	31.70	5.77	32	5632	63	11088	2	352	3	528	0	0	6.40
RM 13	6	15.90	5.54	10.80	34.5	62	19.40	31.10	6.10	38	6042	59	9381	2	318	1	159	0	0	6.30
	8	17.30	5.68	10.10	33.5	59	17.80	30.20	7.37	33	5709	57	9861	4	692	6	1038	0	0	6.90
	14	15.90	6.36	9.10	31.8	50	14.40	28.80	8.37	40	6360	55	8745	0	0	5	795	0	0	6.80
	0	10.70	4.95	12.40	37.3	75	25.00	33.20	4.44	32	3424	56	5992	2	214	10	1070	0	0	6.20
	4	10.50	5.25	12.70	38.7	74	24.20	32.90	2.07	32	3360	64	6720	0	0	4	420	0	0	6.10
RM 14	6	6.90	4.41	10.50	32.3	73	23.90	32.70	1.62	32	2208	62	4278	2	138	4	276	0	0	6.00
	8	11.60	5.10	12.30	37.5	74	24.10	32.80	5.50	34	3944	58	6728	6	696	2	232	0	0	6.00
	14	11.50	5.38	12.70	39.1	73	23.60	32.50	4.38	33	3795	63	7245	1	115	3	345	0	0	6.50
	0	7.80	5.33	12.90	39.9	75	24.10	32.20	4.40	13	1014	80	6240	0	0	7	546	0	0	6.30
	4	9.00	5.43	12.30	38.8	72	22.70	31.70	4.91	8	720	88	7920	1	90	3	270	0	0	6.30
RM 15	6	7.20	5.51	12.40	39.5	72	22.50	31.30	5.19	29	2088	71	5112	0	0	0	0	0	0	6.60
	8	9.20	5.33	12.10	38.2	72	22.60	31.60	5.41	16	1472	79	7268	4	368	1	92	0	0	6.40
	14	9.90	5.55	12.40	39.7	71	22.40	31.30	4.28	13	1287	82	8118	2	198	3	297	0	0	6.70
	0	5.60	5.66	13.50	41.5	73	23.80	32.50	2.73	22	1232	74	4144	4	224	0	0	0	0	6.60
	4	6.90	5.67	13.00	41.0	72	23.00	31.80	3.25	23	1587	74	5106	1	69	2	138	0	0	6.60
RM 16	6	5.90	5.29	12.30	38.6	73	23.30	31.90	4.76	19	1121	77	4543	0	0	4	236	0	0	6.70
	8	5.60	5.28	12.30	38.8	73	23.20	31.60	5.06	21	1176	71	3976	5	280	3	168	0	0	6.80
	14	4.70	5.82	13.30	41.8	72	22.20	31.70	4.23	18	846	76	3572	1	47	5	235	0	0	7.20

Abbreviations:

- WBC: white blood cells
- RBC: red blood cells
- Hgb: hemoglobin
- HCT: hematocrit
- MCV: mean corpuscular volume
- MCH: mean corpuscular hemoglobin
- MCHC: mean corpuscular hemoglobin concentration
- Plts: platelets
- Segm: segmented neutrophils
- Lympho: lymphocytes
- Mono: monocytes
- Eos: eosinophils
- Baso: basophils

Table S2. Reagents for flow cytometric analysis

Assay	Target	Clone	Fluorochrome	Vendor	Catalogue No.	Titration
**Spike-Specific B Cells**						
	Viability	N/A	Aqua	Invitrogen	L34966	1:1000
	CD3	SP34-2	APC-Cy7	BD	557757	1:20
	CD8	RPA-T8	PE-CF594	BD	562282	1:20
	CD14	M5E2	BV786	BD	563698	1:20
	CD16	3G8	PE-Cy7	BD	560716	1:20
	CD20	2H7	BUV395	BD	563782	1:20
	CD27	O323	PerCPeFluor710	Invitrogen	46-0279-42	1:20
	IgD	Polyclonal	AF488	Southern Bio	2030-30	1:20
	IgM	G20-127	BV421	BD	562816	1:20
	S-specific	N/A	APC	N/A	N/A	2 µg
	S-specific	N/A	PE	N/A	N/A	2 µg
**T Cell Cytokines**						
	Viability	NA	Aqua	Invitrogen	L34966	1:1000
	CD3	SP34-2	APC-Cy7	BD	557757	1:50
	CD4	L200	PE-CF594	BD	562402	1:50
	CD8	RPA-T8	BV786	BD	563823	1:50
	CD45RA	5H9	V450	BD	561220	1:50
	CCR7	3D12	PE-Cy7	BD	557648	1:25
	IL-2	MQ1-17H12	PerCP-Cy5.5	BD	560708	1:25
	IL-17	eBio64CAP17	PE	eBiosciences	12-7178-42	1:25
	IFN-g	B27	Alexa F 700	BD	557995	1:25
	TNF-a	Mab11	APC	BD	551384	1:25
	Granzyme B	GB11	FITC	BD	560211	1:25
**Activation-Induced Marker**						
	Viability	N/A	Aqua	Invitrogen	L34966	1:1000
	CD3	SP34-2	BUV395	BD	564117	1:20
	CD4	L200	FITC	BD	566802	1:20
	CD8	RPA-T8	BV786	BD	563823	1:20
	CD20	2H7	APC-H7	BD	560734	1:20
	CD183	1C6/CXCR3	BB700	BD	566532	1:10
	CD185	MU5UBEE	PE-Cy7	Invitrogen	25-9185-42	1:20
	CD196	11A9	PECF594	BD	564816	1:10
	CD134	L106	PE	BD	340420	1:5
	CD137	4B4-1	BV650	Biolegend	309828	1:20
	CD279	EH12.2H7	BV421	Biolegend	329920	1:20
**Germinal Center B & T Cells**						
	Viability	N/A	Aqua	Invitrogen	L34966	1:1000
	CD3	SP34-2	BUV395	BD	564117	1:25
	CD4	L200	FITC	BD	566802	1:25
	CD8	RPA-T8	BV786	BD	563823	1:25
	CD20	2H7	APC-H7	BD	560734	1:25
	CD27	O323	PerCP-eFluor710	Invitrogen	46-0279-42	1:20
	CD185	MU5UBEE	PE-Cy7	Invitrogen	25-9185-42	1:25
	CD279	EH12.2H7	BV650	Biolegend	329950	1:25
	Ki-67	B56	BV421	BD	562899	1:20
	IL-21	3A3-N2.1	PE	BD	560463	1:5
	Bcl-6	K112-91	PE-CF594	BD	562401	1:20
	IL-4	8D4-8	APC	Invitrogen	17-7049-42	1:20
